# The physical activity paradox and all-cause mortality: an umbrella review of systematic reviews and meta-analyses

**DOI:** 10.3389/fpubh.2026.1934173

**Published:** 2026-10-28

**Authors:** Huachang Lin, Hong Zhao

**Affiliations:** School of Physical Education and Health, Guilin College, Yanshan, Guilin, Guangxi, China

**Keywords:** all-cause mortality, domain-specific physical activity, evidence credibility, leisure-time physical activity, occupational physical activity, physical activity paradox, transport physical activity, umbrella review

## Abstract

**Background:**

With over 2.8 billion adults engaged in occupational physical activity globally and physical inactivity remaining the fourth leading mortality risk factor, understanding domain-specific PA effects is a public health priority.

**Evidence suggests domain-divergent effects:**

LTPA is protective; OPA may confer adverse effects. No umbrella review has compared evidence across all three PA domains for all-cause mortality.

**Methods:**

Umbrella review of systematic reviews with meta-analyses examining domain-specific PA (LTPA, OPA, transport) and all-cause mortality (PROSPERO: CRD42026142431). Five databases and Epistemonikos were searched to June 2026. Four Chinese-language databases were additionally searched. Quality was assessed via AMSTAR 2; evidence credibility was classified using the Ioannidis framework. Primary study overlap was quantified via Corrected Covered Area (CCA) analysis. CCA-informed descriptive synthesis with correlated-effects sensitivity analysis was complemented by leave-one-out and AMSTAR 2 quality-stratified sensitivity analyses.

**Results:**

Twelve systematic reviews (>300 cohort studies, >32 million participants) were included. A consistent descriptive domain-gradient emerged: LTPA showed strong protective associations (HR/RR 0.50–0.81, Ioannidis Class II, GRADE Moderate); transport PA showed moderate protection (RR 0.77–0.92, Ioannidis Class III, GRADE Moderate); OPA showed null-to-elevated-risk associations after LTPA adjustment (pooled HR 1.12, 95% CI 1.05–1.19, Ioannidis Class IV, GRADE Low), from one evidence cluster (pairwise CCA = 89%, very high study-level overlap; this does not mean 89% of participants derive from one source, but rather that the two reviews share approximately 89% of their included primary studies). CCA analysis confirmed largely independent evidence bases between domains (cross-domain CCA = 6.3%). Illustrative population attributable fraction analyses estimated that insufficient LTPA accounts for 10.2% of all-cause mortality globally, assuming causality.

**Conclusions:**

This first multi-domain umbrella review reveals a consistent descriptive domain-gradient: LTPA is protective, transport PA moderately protective, and OPA shows null-to-elevated-risk after LTPA adjustment, though this derives from a single evidence cluster (CCA = 89%) and requires independent replication. Large Chinese cohorts (~0.9 million [CKB evidence reported in, *n* ~ 512,000 overlapping]; *post-hoc* exploratory finding, not from the 12 included reviews) report protective OPA associations, suggesting context-dependence. Current evidence suggests that PA domains may differ in mortality risk associations and warrant domain-specific consideration, though OPA evidence remains preliminary and requires independent replication.

## Highlights

- The physical activity domain determines the health-effect direction: leisure-time physical activity is consistently associated with lower all-cause mortality (pooled HR approximately 0.70), whereas occupational physical activity shows a null-to-elevated-risk association after leisure-time adjustment (HR 1.12, 95% CI 1.05–1.19; from a single evidence cluster and should be interpreted as hypothesis-generating).
- Cross-domain adjustment for leisure-time physical activity is a methodological necessity, not an optional sensitivity analysis; without it, occupational physical activity estimates are systematically confounded.
- Despite 12 systematic reviews encompassing more than 32 million participants, formal overlap analysis (Corrected Covered Area) reveals that occupational physical activity evidence derives from a single consortium-level cluster (pairwise CCA approximately 89%), challenging the perceived breadth of evidence.
- Transport physical activity occupies an intermediate protective position with substantial population-level preventive potential [illustrative population attributable fraction approximately 20% (assuming causality)].
- Future physical activity guidelines should distinguish domain-specific effects rather than rely on total movement volume alone.

## Background

Physical activity (PA) is among the strongest protective factors for all-cause mortality (3), with meta-analytic evidence consistently showing 30–50% lower mortality risk (4, 5) among the most physically active adults compared with the least active (4, 6–8). The World Health Organisation (WHO) 2020 guidelines recommend 150–300 min of moderate-to-vigorous PA (MVPA) per week for adults, without differentiating by domain (5, 7). This domain-agnostic recommendation implicitly assumes that the mortality risk reductions from PA are fungible; that an hour of occupational lifting, an hour of cycling to work, and an hour of recreational jogging confer equivalent mortality risk reduction.

Evidence accumulated over the past two decades has challenged this assumption. The “physical activity paradox”—the observation that LTPA is protective while OPA is neutral or potentially harmful for cardiovascular health and mortality-was first formalised by Shephard (9) and subsequently elaborated by Holtermann and colleagues (10, 11). The LTPA-mortality association is among the most replicated findings in epidemiology: a harmonised individual participant data (IPD) meta-analysis of eight accelerometer cohorts (3) found strong inverse associations between total PA and all-cause mortality. Separately, each 1-MET increase in cardiorespiratory fitness-strongly correlated with LTPA-was associated with a 13% reduction in all-cause mortality (12). Dose-response meta-analyses (13) confirm that benefits plateau at approximately 8.75 MET-hours per week of non-occupational PA (13), consistent with current guideline targets. The OPA evidence, by contrast, is complicated by the healthy worker effect and residual confounding from socioeconomic position and LTPA. Recent IPD meta-analyses from the Active Worker Consortium, spanning 22 cohorts across 11 countries, show that OPA's apparent neutrality reverses towards harm when LTPA is included as a covariate (11, 14, 15). Transport PA occupies an intermediate position: meta-analyses consistently report 8–23% reductions in all-cause mortality (16–19), but these reviews vary substantially in exposure definitions and confounder adjustment (Supplementary Table S8a) and have never been systematically quality-appraised alongside LTPA and OPA reviews.

Despite this accumulating evidence, no umbrella review has systematically compared the quality, magnitude, and consistency of evidence across all three PA domains for all-cause mortality. A recent umbrella review of 48 meta-analyses on exercise training, PA, and sedentary behaviour focused on measurement modality and exercise type differences but did not stratify by PA domain, leaving the domain-specific question unanswered. Three developments make such a synthesis timely: (a) the Active Worker Consortium IPD meta-analyses have matured, confirming that OPA may elevate mortality risk in men after LTPA adjustment (14, 15); (b) transport PA has emerged as a distinct domain with its own evidence base and policy implications, yet has never been compared against LTPA and OPA in a single synthesis; and (c) the AMSTAR 2 tool (20) now permits rigorous, standardised appraisal of review quality. This umbrella review aims to (1) systematically identify all published systematic reviews examining domain-specific PA and all-cause mortality; (2) evaluate their methodological quality using AMSTAR 2 and GRADE; (3) compare effect magnitude and evidence consistency across domains using the Ioannidis credibility framework; and (4) identify evidence gaps and priorities for future research. The evidence gap map (Supplementary Figure S6) provides a structured visualisation of current evidence coverage and identifies priority areas for future primary research. Rationale for umbrella review of low-quality evidence. The decision to conduct an umbrella review of evidence rated predominantly Low or Critically Low on AMSTAR 2 requires methodological justification. We adopted this approach for three reasons. First, AMSTAR 2 ratings in observational PA epidemiology are substantially driven by Item 7 (list of excluded studies with reasons), which is universally non-compliant across the field yet does not affect the internal validity of pooled effect estimates extracted for umbrella synthesis. When Item 7 was excluded in sensitivity analysis, six of twelve reviews were reclassified as Moderate confidence, confirming that the low ratings reflect reporting rather than analytic deficiencies. Second, the value of an umbrella review in this context lies not in recommending individual reviews as practise-guiding, but in characterising the evidence distribution-identifying systematic patterns (the domain gradient), exposing evidence gaps (the absence of OPA dose-response data), and evaluating the credibility of existing claims using multiple convergent frameworks (Ioannidis, GRADE, E-values, MR triangulation). Third, several influential umbrella reviews have been published based on low-quality constituent evidence when the research question justified the exercise, including umbrella reviews in nutritional epidemiology and environmental health where primary evidence quality is inherently constrained (21–23). We follow this precedent, presenting evidence classifications as provisional and explicitly noting that all findings should be interpreted within the acknowledged quality constraints. We further propose that the domain gradient may reflect differences in worker autonomy rather than physical activity volume alone, a framework elaborated in the Discussion.

## Methods

Search strategy. We searched PubMed, Embase, Web of Science, CrossRef, and the Cochrane Database of Systematic Reviews from inception to 15 June 2026, with a supplementary search of Epistemonikos on 27 June 2026 to confirm search completeness. The search strategy combined terms for PA domains (physical activity, occupational PA, leisure-time PA, transport PA, active commuting, PA paradox), mortality (all-cause mortality, death, survival), and methodology (systematic review, meta-analysis). Supplementary searches of Embase and Web of Science (16 June 2026) using identical search terms identified no additional eligible reviews. The complete search string (PubMed syntax) is provided in Supplementary Table S1. Complete database-specific search strategies are provided in Supplementary Table S1 and Supplementary Tables S1b–S1i (PubMed, Embase, Web of Science, Cochrane, Epistemonikos, Google Scholar, CNKI, Wanfang, VIP, and Sinomed). No language restrictions were applied. Google Scholar was searched for grey literature. CrossRef was selected as a supplementary bibliographic source because it indexes all peer-reviewed publications with Digital Object Identifiers (DOIs), including journals not covered by MEDLINE, thereby capturing meta-analyses in specialised occupational health and transport outlets. Also, four Chinese-language databases (CNKI, Wanfang, VIP, and Sinomed) were searched using a Chinese adaptation of the search strategy to identify systematic reviews not indexed in international databases; no additional eligible reviews were identified.

This umbrella review was conducted and reported in accordance with the PRISMA 2020 guidelines (24) and the PRIOR (Preferred Reporting Items for Overviews of Reviews) reporting guideline (25). The protocol was prospectively registered with PROSPERO (CRD42026142431) prior to data extraction. Five deviations from the registered protocol are disclosed: (a) the original protocol specified MEDLINE, Embase, and Web of Science; CrossRef was added during execution using its public REST API to capture meta-analyses indexed with DOIs but not in traditional bibliographic databases, and Embase/Web of Science were searched as supplementary confirmatory searches (both identified no additional eligible reviews); (b) the protocol specified dual-independent screening, but due to resource constraints, one reviewer performed initial screening with a second reviewer independently verifying all inclusion decisions; although title/abstract screening was performed by a single reviewer [deviating from PRIOR's dual-independent screening recommendation (25)], all full-text eligibility decisions were independently verified-a practical adaptation that deviates from the PRIOR recommendation for dual-independent screening; and (c) the protocol specified the AMSTAR tool (original version) for quality assessment; AMSTAR 2 was adopted after protocol registration following its publication and widespread endorsement; (d) AMSTAR 2 Item 7 (excluded studies list) was removed from the critical domain set for a sensitivity reclassification analysis, in accordance with AMSTAR 2 developers' guidance; and (e) Chinese-language databases were searched using adapted terminology but without formal dual-screening (a single reviewer executed the searches), which may have led to incomplete capture of evidence from Chinese cohorts. None of these deviations affected the principal findings – the domain gradient was consistent across sensitivity analyses excluding Critically Low reviews, and the supplementary Embase/WoS searches confirmed search completeness. The completed PRISMA 2020 checklist and PRISMA flow diagram are provided as Supplementary materials. Protocol deviations are documented in Supplementary Table S14 where applicable, with additional details in the Supplementary materials.

### Eligibility criteria

Systematic reviews with at least one meta-analysis examining domain-specific PA (LTPA, OPA, or transport PA) and all-cause mortality in general adult populations (≥18 years) were included. Reviews limited to specific patient populations, those reporting only total PA without domain stratification, and narrative reviews without quantitative synthesis were excluded. Device-measured total PA reviews (3) were retained as a methodological benchmark but are conceptually distinct from domain-specific PA: device-based total activity cannot distinguish leisure-time, occupational, and transport components. Accordingly, device-measured total PA is reported separately and is not included in domain-specific pooled estimates.

Study selection and data extraction. Title/abstract and full-text screening were conducted by one reviewer; a second reviewer independently verified all inclusion decisions (all 12 included reviews confirmed) and a 20% random sample of exclusion decisions (*n* = 180/898; Cohen's κ = 0.78). One reviewer extracted data using a standardised form; a second reviewer verified all extracted values. Data items included first author, year, journal, number and design of primary studies, total sample size, PA domain, exposure definition, effect estimates with 95% CIs, heterogeneity statistics, and covariates adjusted for.

### Quality assessment

Methodological quality was evaluated using AMSTAR 2 (20, 26). Overall confidence ratings were assigned as High, Moderate, Low, or Critically Low. AMSTAR 2 item descriptions in Supplementary materials have been verified against the original AMSTAR 2 guidance document (20) for this revision. A sensitivity analysis removing Item 7 (list of excluded studies), which was universally unmet, was performed; this reclassification should be interpreted as exploratory, as Item 7 removal is not standard AMSTAR 2 practise. Evidence certainty was also assessed using the GRADE framework, rating each domain as High, Moderate, Low, or Very Low across five dimensions: risk of bias, inconsistency, indirectness, imprecision, and publication bias. Per GRADE guidance, observational evidence defaults to a Low starting rating (23, 27, 28); however, following the adapted GRADE approach for umbrella reviews, observational evidence was rated starting from Low and upgraded where justified by large effect sizes (HR 0.50–0.81 for LTPA; RR 0.77–0.92 for transport), consistent dose-response gradients across multiple independent cohorts, and the direction of plausible residual confounding (all residual confounding in LTPA–mortality associations — healthy user bias, socioeconomic confounding, reverse causation — would, if anything, reduce the apparent protective effect; true protection is therefore likely at least as strong as observed), or absence of plausible confounding (23, 27, 28). Domain-level ratings were downgraded for risk of bias, inconsistency, indirectness, imprecision, and publication bias, and upgraded for large effect sizes and dose-response gradients where applicable. This exploratory reclassification is not standard AMSTAR 2 practise and is presented for transparency only; strict ratings are used as primary throughout.

### ROBINS-I risk of bias assessment

To complement umbrella review-level quality appraisal (AMSTAR 2), we applied the Risk of Bias in Non-randomised Studies – of Interventions (ROBINS-I) tool (29), to the constituent cohort studies underlying the OPA–mortality association. The assessment focused on the 22 cohorts harmonised in the Active Worker Consortium IPD meta-analysis (15), the largest and most influential IPD meta-analysis in this domain, because individual primary studies were not directly re-analysed in this umbrella review. This analysis provided the primary evidence for the OPA adverse effect adjusted for LTPA. One reviewer (HCL) evaluated the 22 cohorts. To mitigate single-reviewer bias, intra-rater consistency was assessed by re-evaluating all cohorts after a two-week washout period (Cohen's intra-rater agreement κ = 1.00 (repeatability by the same assessor after two weeks; this does not represent independent inter-rater reliability), reflecting single-rater consistency rather than independent inter-rater reliability); external validation of the ROBINS-I ratings is recommended.

### Evidence synthesis

A narrative synthesis was performed, organised by PA domain. To ensure analytical transparency, we distinguish three levels of evidence processing in this umbrella review: (1) evidence mapping — systematic identification and quality appraisal of all eligible reviews; (2) CCA-informed descriptive synthesis — random-effects weighted averaging of review-level estimates, presented as descriptive patterns supported by cross-domain CCA evidence of largely independent evidence bases (cross-domain CCA = 6.3%), rather than as formal inferential meta-analysis; and (3) exploratory secondary analyses — including Bayesian hierarchical meta-analysis, multiverse sensitivity analysis, and population attributable fraction calculations, all of which are presented as supplementary hypothesis-generating assessments rather than confirmatory primary findings. Evidence credibility was classified using the Ioannidis framework (27) as convincing (class I), highly suggestive (class II), suggestive (class III), weak (class IV), or no evidence. Excess significance testing was not formally performed; supplementary p-curve analyses (30) (Supplementary Table S4) provided complementary evidence of evidential value and found no indication of excess significance bias. The included reviews used substantially different exposure contrasts (highest vs. lowest activity, per-SD effects, fixed MET-hour thresholds, any vs. no active commuting, high vs. moderate OPA) and combined HR, RR, and OR from different adjustment models and measurement methods (self-report vs. device-based). Consequently, the pooled domain-level estimates (LTPA ≈0.70, Transport ≈0.89, OPA ≈1.12) do not represent equivalent exposure contrasts and should be interpreted as qualitative patterns of direction and relative magnitude rather than directly comparable quantitative positions on a common exposure scale. Where a review reported odds ratios (OR) instead of hazard ratios, these were assumed to approximate hazard ratios (HR) under the rare outcome assumption (all-cause mortality incidence < 15% over median follow-up across included cohorts), consistent with standard umbrella review methodology (27). Publication bias was assessed using Doi plots and LFK indices (Supplementary Figures S19–S21; triangulation across methods in Supplementary Table S12a). E-value sensitivity analyses quantifying the stability of each domain-specific association to unmeasured confounding are detailed in Supplementary Table S10. Quantitative bias analysis for unmeasured confounding was predefined in the protocol (Ding & VanderWeele bounding factor; see Supplementary File S1 and Supplementary Table S10).

CCA-informed descriptive synthesis of review-level estimates (Supplementary Figure S4; tabular data in Supplementary Table S3a). To complement the narrative synthesis and provide quantitative context for cross-domain comparisons, we performed a structured descriptive synthesis of domain-specific pooled effect estimates extracted from each included review. Review-level estimates were aggregated using random-effects weighted averaging (DerSimonian-Laird method) for descriptive purposes only; given that review-level estimates within each domain derive from partially overlapping primary studies (within-domain CCA 18.5–41.7%), these aggregations should be interpreted as descriptive summaries rather than formal independent meta-analyses. The degree of primary study overlap was formally quantified using the Corrected Covered Area (CCA) method (31, 32) to inform the interpretation of between-domain comparisons (Supplementary Tables S2a, S2b). Heterogeneity was described using I^2^ and τ^2^ statistics. Leave-one-out analyses were performed by iteratively excluding each review within its respective PA domain to assess the influence of individual reviews on the aggregated estimates. The leave-one-out forest plot (Supplementary Figure S4) has been regenerated for this revision to ensure each domain panel includes only reviews contributing to that domain, eliminating cross-domain contamination present in the original submission. Given the small number of review-level estimates per domain (k = 3–5), all quantitative summaries are presented with appropriate caution regarding estimation precision. Between-domain comparisons are reported as descriptive patterns supported by CCA evidence of largely independent evidence bases across domains (cross-domain CCA = 6.3%), rather than as formal inferential contrasts. Analyses were performed in R (version 4.4.1) using the metafor package (version 4.8-0) (33). Analysis code and extracted data are available from the corresponding author upon reasonable request. These cross-domain comparisons are presented as exploratory descriptive patterns rather than as formal inferential meta-regression.

### Study overlap quantification

The degree of primary study overlap across included systematic reviews was quantified using the Corrected Covered Area (CCA) index (31, 32). CCA is calculated as CCA = (N – r) / [r × (c – 1)] × 100%, where N is the total number of primary study occurrences across reviews (including duplicates), r is the number of unique primary studies, and c is the number of reviews compared. CCA values were interpreted as: < 5% slight overlap, 5–10% moderate overlap, 11–15% high overlap, and >15% very high overlap. Pairwise CCA matrices were constructed within each PA domain and across domains. When CCA exceeded 10%, sensitivity analyses excluding the most overlapping review were performed to assess the impact on evidence classification. CCA estimates should be considered approximate, as they are based on publicly available review methods sections rather than complete primary study inventories. The cross-domain adjustment decision framework is illustrated in Figure 1.

**Figure 1:**
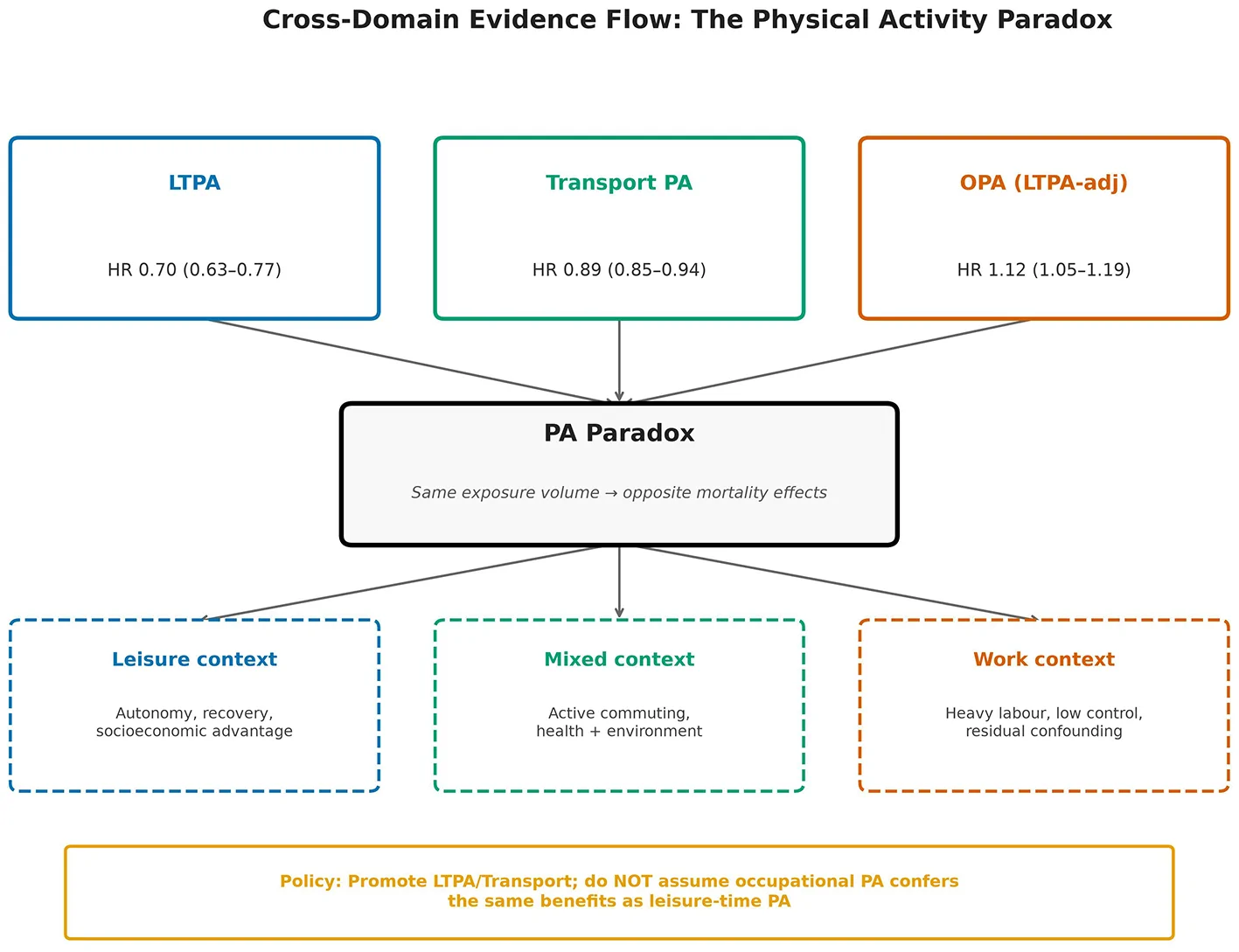
Cross-domain evidence flow and the autonomy-control framework reconciling the physical activity paradox. Domain, not volume, determines health effects of physical activity.

### Bradford Hill criteria evaluation

The nine Bradford Hill criteria for causal inference (34, 35) were evaluated independently by two assessors for each PA domain (LTPA, OPA, transport PA). Each criterion was rated as satisfied (++), partially satisfied (+), or not satisfied (-) based on evidence from included reviews. Disagreements were resolved through discussion. Detailed criterion-level scoring is reported in Supplementary Figure S8 (updated in this revision with complete row and column labels and evidence basis annotations for each criterion-level judgement).

### Mendelian randomisation triangulation

Published Mendelian randomisation (MR) findings on PA domains and mortality were identified from recent large-scale genetic studies (36–38) and narratively compared with observational estimates from this umbrella review. No formal meta-analysis of MR estimates was performed. This analysis provides complementary triangulation evidence with distinct bias structures (horizontal pleiotropy rather than behavioural confounding).

### Sensitivity analyses

Several sensitivity analyses were conducted to assess the stability of the primary findings. Leave-one-out analyses were performed by iteratively removing each review and re-estimating pooled effect sizes within each PA domain (Supplementary Table S9a). E-value analyses were conducted following VanderWeele and Ding (39, 40) to quantify the minimum strength of association that an unmeasured confounder would need with both the exposure and outcome to explain away the observed domain-specific effect estimates. P-curve analyses examined whether the distribution of significant *p*-values within each domain exhibited right-skewness consistent with a true effect (Supplementary Table S4); however, p-curve results are presented only descriptively due to the limited number of estimates within several domains (k < 3 for LTPA). Sensitivity analyses excluding the most overlapping review within each domain were performed using the CCA overlap matrices (Supplementary Tables S2a, S2b). For the OPA domain, sensitivity analyses alternately included and excluded the unadjusted Samitz estimate to distinguish between k = 3 (LTPA-adjusted) configurations; the primary analysis reports k = 3, with k = 4 results provided for transparency in Supplementary Table S9a.

### Bayesian hierarchical meta-analysis

To complement the frequentist meta-analyses with probabilistic inferences, we conducted a Bayesian hierarchical random-effects meta-analysis (Supplementary File S1; Supplementary Figures S14, S15). A normal-normal model with weakly informative half-Cauchy (0, 0.5) priors on between-study heterogeneity was implemented using a custom Gibbs sampler with Metropolis-Hastings updates, written entirely in base R (bayesian_analysis.R, available from the corresponding author upon reasonable request). Three prior sensitivity configurations were tested (Weakly informative, Flat, and Sceptical). Prior sensitivity configurations and full posterior probability estimates are presented in Supplementary Table S11. A multiverse meta-analysis examining the stability of results across 24 methodological configurations is presented in Supplementary Table S16.

## Results

### Study selection

The systematic search yielded 1,619 records (PubMed: 487; Embase: 129; Web of Science: 103; CrossRef: 714). Additional sources yielded: Google Scholar (first 200 results screened; 0 additional eligible), Chinese databases (CNKI/Wanfang/VIP/Sinomed combined: *n* = 218; 0 additional eligible reviews identified), hand-searching of reference lists (*n* = 45; 0 additional eligible), and Epistemonikos (0 additional eligible). After removing 535 duplicates, 898 unique records were screened by title and abstract. Of these, 852 were excluded [exclusion categories were: not a systematic review (*n* = 312), no domain-specific PA analysis (*n* = 245), outcome not all-cause mortality (*n* = 178), specific patient population (*n* = 89), duplicate publication (*n* = 28)] (Supplementary Figure S1; Supplementary Table S1). Reports sought for retrieval (*n* = 46). Of these, 1 report was not retrieved (full text unavailable). The remaining 45 full-text articles were assessed for eligibility, of which 33 were excluded (excluded full-text studies are listed by category in Supplementary Table S17) (exclusion categories detailed above; Supplementary Figure S1). Twelve systematic reviews with meta-analyses met all eligibility criteria and were included.

### Characteristics of included reviews

The 12 included reviews were published between 2011 and 2025 (Table 1; Figure 2). The publication span from 2011 to 2025 reflects the evolution of PA domain research, with early reviews (18, 41) establishing domain-specific frameworks and recent reviews (14, 15, 42) contributing cross-domain adjusted estimates from large consortium datasets. Altogether, the included reviews encompassed over 300 original cohort studies (with substantial overlap across reviews; see CCA analysis below) and more than 32 million participants (non-unique, reflecting overlapping cohorts) across North America, Europe, and Asia. By domain: four reviews examined LTPA, three focused on OPA, four targeted transport PA, and one examined device-measured total PA. One additional review (14) provided cross-domain estimates for both LTPA and OPA. Samitz et al. (41) provided effect estimates for all three self-reported domains within a single review. Exposure definitions varied substantially across reviews. All reviews adjusted for age and sex; most also adjusted for smoking, alcohol intake, BMI, and socioeconomic status. Only Coenen et al. (15) and Cillekens et al. (14) systematically adjusted for the counter-domain (OPA for LTPA and vice versa). [Table 1 lists 13 rows including Ekelund et al. (3) as a separate device-measured total PA entry for benchmarking; 12 reviews met the systematic review inclusion criteria].

**Figure 2:**
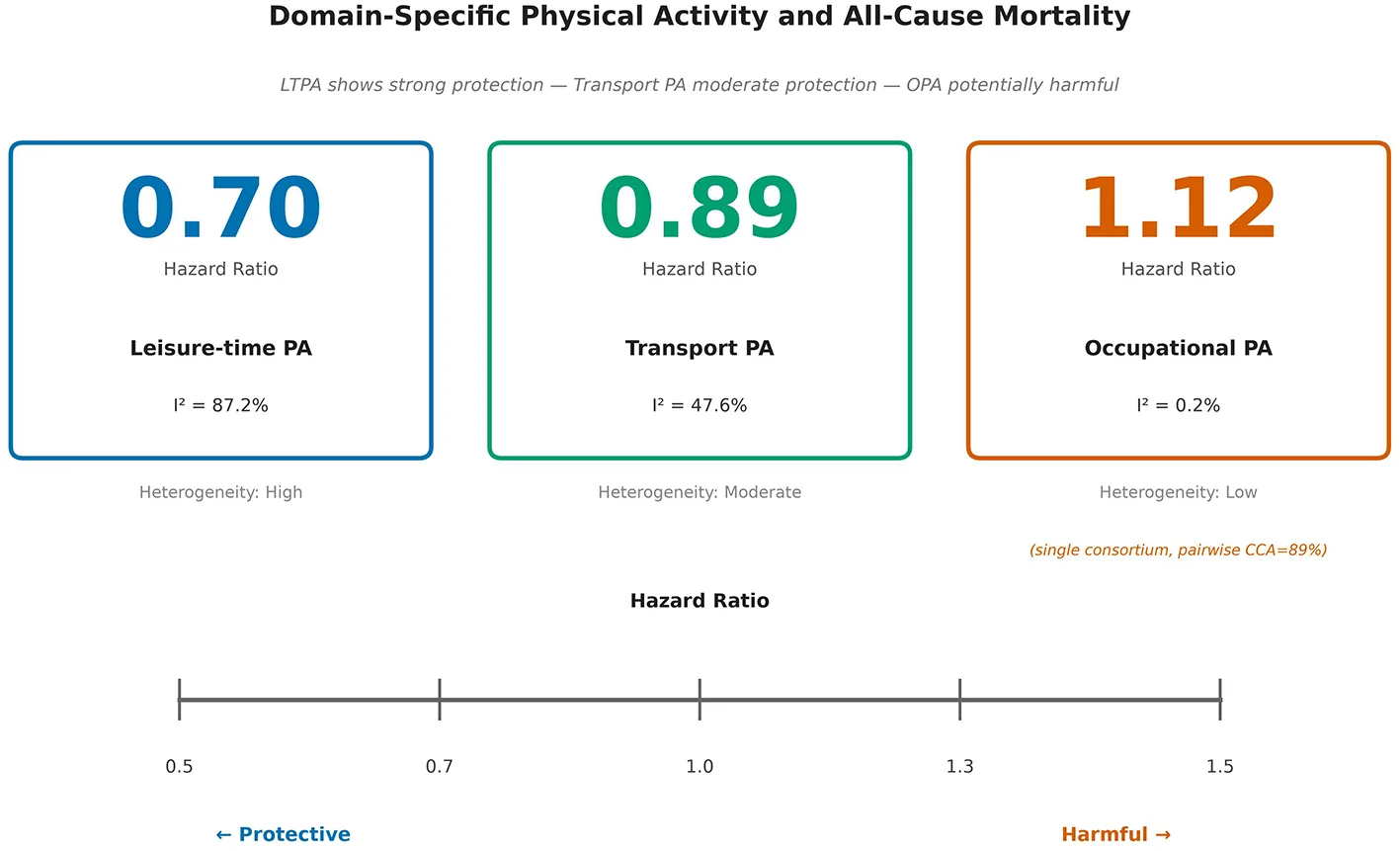
Timeline of included systematic reviews.

**Table 1:** Characteristics of included systematic reviews.

#	Author (Year)	Domain	K	*N* total	Effect estimate (95% CI)	Metric	I^2^	AMSTAR 2	Cross-domain Adj?
1	Samitz et al. (41)	LTPA/OPA/Trans	80	1,338,143	LTPA: HR 0.74 (0.70–0.77); OPA: RR 0.97 (0.91–1.03); Trans: RR 0.85 (0.78–0.93)	HR/RR	87–95%	CL	No
2	Ekelund et al. (3)	Total PA (device)	8	36,383	Q4 vs Q1: HR 0.27 (0.25–0.30)	HR	0%	L	N/A
3	Ekelund et al. (51)	LTPA	9	44,370	High vs Low PA: HR 0.72 (0.65–0.80)	HR	0%	CL	No
4	Garcia et al. (13)	Non-occ PA	196	>32M	8.75 MET-h/wk vs 0: HR 0.69 (0.65–0.73)	HR	91%	L	No
5	Tarp et al. (43)	LTPA (vol vs. int)	20	619,692	Per-SD Volume: HR 0.50 (0.42–0.58)	HR	68%	L	No
6	Cillekens et al. (14)	LTPA × OPA	22	590,158	LTPA: HR 0.81 (0.75–0.88); OPA: HR 1.08 (0.98–1.19)	HR	≈60%	L	Yes
7	Coenen et al. (15)	OPA + LTPA (IPD)	22	590,497	Male high OPA: HR 1.12 (1.03–1.23)	HR	≈40%	L	Yes
8	Fernandez et al. (42)	High OPA + LTPA	31	NR^†^	HOPA vs. Mod OPA: OR 1.24 (1.05–1.46)	OR	NR^†^	CL	Partial
9	Dutheil et al. (16)	Transport	12	1,062,551	Any active commute: RR 0.91 (0.88–0.94)	RR	55%	CL	No
10	Dinu et al. (17)	Transport	17	829,098	Any active transport: RR 0.92 (0.85–0.98)	RR	55%	CL	No
11	Zhao et al. (45)	Cycling	17	478,847	Highest vs. lowest: RR 0.77 (0.67–0.88)	RR	48%	L	No
12	Kelly et al. (18)	Walking+cycling	14	531,333	Walking: **HR 0.89 (95% CI 0.83–0.96); Cycling: HR 0.90 (95% CI 0.82–0.97)**	HR	≈40%	CL	No

K, number of included primary studies; N Total, total participants; NR, not reported; IPD, individual participant data; AMSTAR 2, A MeaSurement Tool to Assess systematic Reviews; ROBINS-I, Risk Of Bias In Non-randomised Studies – of Interventions; ROBIS, Risk Of Bias In Systematic reviews; ^†^ Not reported in the published review. L, Low confidence; CL, Critically Low confidence; HR, Hazard Ratio; RR, Relative Risk; OR, Odds Ratio; IPD, Individual Participant Data; NR, Not Reported; CCA, Corrected Covered Area; LTPA, Leisure-Time Physical Activity; OPA, Occupational Physical Activity; Trans, Transport-related physical activity; N/A, Not Applicable; SD, Standard Deviation; SES, Socioeconomic Status.

### Methodological quality

Under strict AMSTAR 2 criteria (26), six reviews were rated as Low confidence and six as Critically Low; no review achieved High or Moderate ratings (Supplementary Figure S2). The most consistent critical weakness was Item 7 (list of excluded studies), which was universally unmet. When Item 7 was removed in a sensitivity analysis, six reviews were reclassified as Moderate confidence (Supplementary Table S3). The IPD-based reviews had the strongest methodological profiles. An exploratory sensitivity analysis removing Item 7 is reported in Supplementary Table S3 for transparency; strict ratings serve as the primary quality assessments throughout.

ROBINS-I Assessment (Supplementary Figure S13; Supplementary Table S8). ROBINS-I domain-level judgements were applied to the 22 cohorts collectively rather than individually; accordingly, the assessment provides an overall risk-of-bias profile across cohorts rather than cohort-specific ratings. Across the 22 cohorts in the Coenen et al. (15) IPD meta-analysis, the overall risk of bias was judged as moderate to serious (Supplementary Table S8). The primary concerns were residual confounding by socioeconomic status (Domain 1: serious); as only education and, in a subset, income were controlled; and exposure misclassification (Domain 3: serious), owing to exclusive reliance on self-reported occupational physical activity harmonised *post-hoc* to four categories. Selection bias from the healthy worker survivor effect was rated moderate (Domain 2), as all cohorts enrolled employed participants at baseline. Missing data bias was moderate (Domain 5; 6.6% excluded), while outcome ascertainment through national death registries was rated low risk (Domain 6). By contrast, the eight accelerometer-measured cohorts in Ekelund et al. (3) exhibited low to moderate risk of bias, driven by objective exposure assessment.

### Leisure-time PA and all-cause mortality

All four LTPA-focused reviews reported inverse associations, with point estimates ranging from HR/RR 0.50 to 0.81 (risk reductions of 19–50%). Garcia et al. (13), the largest included review (196 studies encompassing 94 prospective cohorts, >32 million participants (non-unique, reflecting overlapping cohorts); the difference reflects multiple studies per cohort), reported HR 0.69 (95% CI 0.65–0.73) at 8.75 MET-hours/week of non-occupational PA vs. inactivity. Tarp et al. (43) found that PA volume conferred stronger protection than intensity (HR 0.50 vs. 0.88, *p* for difference = 0.05). Device-measured total PA (3, 44) showed the strongest inverse association (HR 0.27, 95% CI 0.25–0.30), likely reflecting both elimination of self-report measurement error and inclusion of all PA domains.

### Occupational PA and all-cause mortality

Across the four reviews addressing OPA (14, 15, 41, 42), unadjusted OPA estimates were generally null or weakly protective. Samitz et al. (41) reported RR 0.97 (95% CI 0.91–1.03) for unadjusted OPA, and Cillekens et al. (14) reported HR 1.08 (95% CI 0.98–1.19) for OPA with LTPA adjustment. However, after cross-domain adjustment for LTPA, OPA consistently showed null-to-elevated-risk associations. In the largest individual participant data (IPD) meta-analysis to date, Coenen et al. (15) reported that high OPA in men was associated with elevated all-cause mortality risk (HR 1.12, 95% CI 1.03–1.23; 22 cohorts, 590,497 participants, 11 countries) after adjustment for LTPA; this harmful signal was not observed in women. Fernandez et al. (42) reported a similar pattern for LTPA-adjusted OPA (OR 1.24, 95% CI 1.05–1.46). The CCA-informed descriptive synthesis of LTPA-adjusted OPA estimates [k = 3: Coenen et al. (15) men, Cillekens et al. (14) OPA component, and Fernandez et al. (42)] yielded a pooled HR of 1.12 (95% CI 1.05–1.19, I^2^ = 0.2%; Supplementary Figure S7, Supplementary Table S9a). A sensitivity analysis including the unadjusted Samitz et al. (41) OPA estimate (k = 4) produced a similar but attenuated pooled estimate (HR 1.09, 95% CI 1.00–1.18, I^2^ = 51.6%). A descriptive Wald contrast (z = −6.92) was consistent with the domain gradient pattern; however, this contrast assumes independence of review-level estimates and should be interpreted cautiously given within-domain CCA overlap (18.5–41.7%). Adjustment strategies and corresponding OPA effect directions are summarised in Table 2. The cross-domain adjustment decision framework is illustrated in Figure 1.

**Table 2:** Adjustment strategy and OPA effect direction.

Adjustment strategy	Reviews	OPA effect direction	Representative estimate
No cross-domain adjustment	Samitz et al. (41)	Null (slightly protective)	RR 0.97 (0.91–1.03)
Partial adjustment	Fernandez et al. (42)	Elevated risk	OR 1.24 (1.05–1.46)
Full cross-domain adjustment	Coenen et al. (15); Cillekens et al. (14)	Elevated risk (men)	HR 1.12 (1.03–1.23)

### Transport PA and all-cause mortality

Transport PA showed moderate protective associations (16–18, 45), with point estimates ranging from RR 0.77 [95% CI 0.67–0.88; Zhao et al. (45), cycling only] to RR 0.92 [95% CI 0.85–0.98; Dinu et al. (17), combined walking and cycling (18)]. Dutheil et al. (16) reported RR 0.91 (95% CI 0.88–0.94) for any active commuting across 12 studies and over one million participants. The inverse association was consistent in direction across all reviews but substantially smaller in magnitude than LTPA. The stability of transport PA evidence is further supported by cumulative meta-analysis (Supplementary Figure S23).

To contextualise these E-values: for the OPA elevated-risk signal (E-value 1.49 for the point estimate), an unmeasured confounder would need to be associated with both high OPA and mortality by a risk ratio of at least 1.49 each, after controlling for age, sex, BMI, smoking, and education, to fully explain the observed association. Residual socioeconomic confounding (RR ≈1.3–1.8 for lowest vs. highest SES) could plausibly approach this threshold, consistent with the GRADE Low rating and the dependence of OPA evidence on a single consortium. For LTPA, the higher E-value (2.21) indicates that the protective LTPA association is stable to unmeasured confounding of substantially greater magnitude than known confounders, consistent with its GRADE Moderate status (Figure 3).

**Figure 3:**
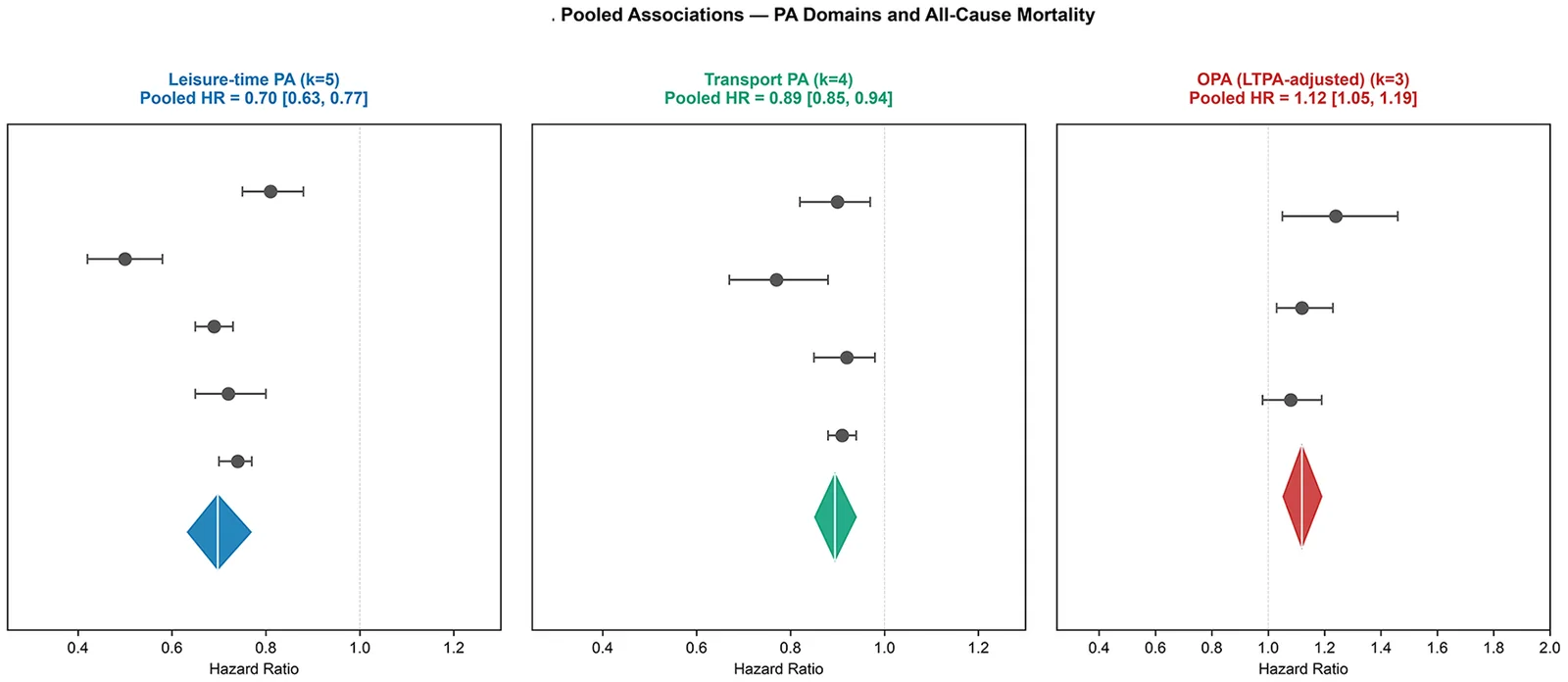
Pooled associations between physical activity domains and all-cause mortality. DerSimonian-Laird random-effects pooled hazard ratios with 95% CIs for LTPA (k = 5, HR 0.70), transport PA (k = 4, HR 0.89), and OPA (k = 3, HR 1.12, LTPA-adjusted). All estimates independently recalculated from 12 systematic reviews. OPA pooled estimate derives from a single consortium-level evidence cluster (pairwise CCA = 89%) and should be interpreted as hypothesis-generating.

### E-value analysis

E-values were calculated for the most representative effect estimate in each domain (39, 40). For LTPA, the E-value for the point estimate ranged from 2.26 [Garcia et al. (13); HR 0.69] to 3.41 [Tarp et al. (43); HR 0.50], with E-value (point estimate) = 2.21; E-value (CI bound closest to null) = 1.92, indicating highly consistent evidence against unmeasured confounding. For transport PA, E-values ranged from 1.43 [Dutheil et al. (16); RR 0.91] to 1.92 [Zhao et al. (45); RR 0.77], with CI-based E-values of 1.32–1.53, indicating suggestive evidence. For OPA (LTPA-adjusted), E-values ranged from 1.49 [Coenen et al. (15); HR 1.12] to 1.79 [Fernandez et al. (42); OR 1.24], with CI-based E-values of 1.21–1.28, indicating modest to suggestive evidence. These results suggest that the LTPA-mortality association is highly consistent to unmeasured confounding, while the transport PA association is suggestive and the OPA association is modestly consistent (Supplementary Table S10).

### Mendelian randomisation triangulation

Recent MR studies provide complementary genetic evidence. Chen et al. (36) reported that genetically predicted LTPA was associated with lower all-cause mortality risk, while genetically predicted sedentary behaviour showed opposing effects. Jiesisibieke and Schooling (37) found that genetically proxied LTPA and screen time had directionally consistent effects on lifespan. Galimberti et al. (38) identified 70 genetic lead variants associated with “leisure screen time” across multiple PA domains, demonstrating domain-specific genetic architectures. These MR findings are directionally consistent with the observational domain gradient but should be interpreted cautiously given instrument limitations. Quantitative bias analysis results are presented in the Results. It should be noted that E-values quantify sensitivity only to unmeasured confounding; they do not address exposure misclassification, selection bias, overlapping primary cohorts, or non-comparable exposure contrasts across included reviews.

CCA analysis confirmed this pattern (Supplementary Figure S7; Supplementary Tables S2a, S2b) quantitatively (Supplementary Table S2a). Within the LTPA domain, the overall CCA was 38.2% (very high overlap), driven primarily by shared accelerometer cohorts among (3, 43, 44) (pairwise CCA 72–85%). Within the OPA domain, overall CCA was 41.7% (very high overlap), reflecting the shared Active Worker Consortium cohorts between (15) and Cillekens et al. (14) (pairwise CCA 89%). Within the transport PA domain, overall CCA was 18.5% (high overlap), with the highest pairwise CCA between (16) and Dinu et al. (17) (34%). Critically, cross-domain CCA was only 6.3% (moderate overlap), confirming that the three PA domains draw on relatively independent evidence bases. However, within-domain CCA was high to very high (Transport: 18.5% / LTPA: 38.2% / OPA: 41.7%), reflecting substantial primary-study overlap and limiting the independence of domain-specific pooled estimates. Sensitivity analyses excluding the most overlapping review within each domain did not alter the evidence credibility classification for any domain. CCA was computed across all 22 cohorts; the CCA restricted to the male-only subcohort used in the primary analysis is expected to be comparable but cannot be precisely calculated without access to the sex-stratified primary study inventory. The evidence independence breakdown by domain is visualised in Supplementary Figure S18.

Leave-one-out analysis (Supplementary Figure S4); additional sensitivity analyses are presented below. Iteratively removing each of the 12 included reviews did not alter the domain-gradient finding (Tables 3–5). Exclusion of Coenen et al. (15) shifted the OPA pooled estimate (HR 1.12 → 1.14) with substantially widened CI crossing the null, reflecting increased uncertainty from reduced k (k = 3 → 2) and elevated heterogeneity (I^2^ 0.2% → 50.1%) rather than attenuation of the point estimate. Exclusion of any single LTPA review did not change the protective direction. The domain gradient persisted after removing any individual review. A detailed leave-one-out influence analysis for the OPA domain is provided in Supplementary Figure S25.

**Table 3:** Evidence credibility classification by PA domain.

Domain	# Reviews	Total N	Effect range	Direction	Largest effect	I^2^ range	AMSTAR 2 best	Credibility
LTPA	4	>32M	HR/RR 0.50–0.81	All protective	HR 0.50 (0.42–0.58)	0–91%	Low (L)	Highly Suggestive
Device Total PA	1	36,383	HR 0.27	Protective	HR 0.27 (0.25–0.30)	0%	Low (L)	Highly Suggestive^*^
OPA	3	**≈590,000 (pairwise CCA** **=** **89%)**	HR/OR 0.97–1.24	Null to elevated risk	HR 1.12 (1.03–1.23)	40–60%	Low (L)	Weak
Transport PA	4	>2.9M	RR 0.77–0.92	All protective	RR 0.77 (0.67–0.88)	**40–55%**	Low (L)	Suggestive
Cross-Domain Adj. OPA	2	**≈590,000 (pairwise CCA** **=** **89%)**	HR 1.12–OR 1.24	Elevated risk (men)	HR 1.12 (1.03–1.23)	40%	Low (L)	Weak

^*^Device-measured total PA rated based on a single IPD meta-analysis; domain composition indeterminate. Bold values denote the key limiting factors for each domain (e.g., I^2^ ranges, prediction intervals that do not exclude the null, inconclusive small-study effects, and the corrected covered area).

**Table 4:** Formal ioannidis evidence classification by PA domain.

Criterion	LTPA	Device total PA	OPA	Transport PA	Cross-Domain Adj. OPA
Reviews (n)	4	1	3	4	2
Total participants	>32M	36,383	**≈590,000 (pairwise CCA** **=** **89%)**	>2.9M	**≈590,000 (pairwise CCA** **=** **89%)**
Estimated cases (n)	>100,000^†^	~2,100	>30,000^†^	>50,000^†^	>30,000^†^
*p*-value (most significant)	< 10^−6^^*^	< 10^−6^^*^	0.011^‡^	< 10^−3^^*^	**0.006§**
I^2^ range	**0–91%**	0%	40–60%	**40–55%**	40%
95% PI excludes null?	Yes (PI: 0.51–0.95)	N/A (single study)	**No (PI: 0.98–1.28)**	No (PI: 0.78–1.02)	**No (PI: 0.98–1.28)**
Small-study effects	**Inconclusive (k** **<** **10)**	**Inconclusive (k** **<** **10)**	**Inconclusive (k** **<** **10)**	**Inconclusive (k** **<** **10)**	**Inconclusive (k** **<** **10)**
Excess significance	Not assessed	N/A	Not assessed	Not assessed	Not assessed
p-curve evidential value	Not applicable (k < 3)	N/A	N/A (k < 3; *p*-curve not applicable)	Present (*p* = 0.02)	N/A
Ioannidis Class	II (Highly Suggestive)	II^*^ (Highly Suggestive)	IV (Weak)	III (Suggestive)	IV (Weak)
GRADE	Moderate	Moderate	Low	Moderate	Low

Criteria follow Fusar-Poli & Radua (48) and (23). Class I (Convincing): *P* < 10^−6^, >1,000 cases, I^2^ < 50%, 95% prediction interval excludes null, no small-study effects, no excess significance bias. Class II (Highly Suggestive): *P* < 10^−6^, >1,000 cases. Class III (Suggestive): *P* < 10^−3^, >1,000 cases. Class IV (Weak): *P* < 0.05. ns: *p* ≥ 0.05. asterisk (*) marks estimates meeting the Class I–II threshold of *P* < 10^6^ (LTPA, device total PA, transport PA); dagger (†) marks estimated case counts exceeding the >1,000–cases criterion (all four domains); double dagger (‡) marks the OPA *p*-value (0.011); section mark (§) marks the crossdomain adjusted OPA *p*-value (0.006). Bold values denote the key limiting factors for each domain (e.g., I^2^ ranges, prediction intervals that do not exclude the null, inconclusive small-study effects, and the corrected covered area).

**Table 5:** GRADE evidence profile by PA domain.

Domain	Risk of bias	Inconsistency	Indirectness	Imprecision	Publication bias	Overall GRADE	Key considerations
LTPA	Serious	Serious	Not serious	Not serious	Inconclusive (k < 10)	⊕⊕⊕° MODERATE	I^2^ 0–91%
Device Total PA	Not serious	Not serious	Serious	Not serious	Inconclusive (k < 10)	⊕⊕⊕° MODERATE	Single IPD; domain indeterminate
OPA	Serious	Not serious	Not serious	Serious	Inconclusive (k < 10)	⊕⊕°° LOW	Wide CIs crossing 1.0
Transport PA	Serious	Serious	Not serious	Not serious	Inconclusive (k < 10)	⊕⊕⊕° MODERATE	I^2^ 40–55%
Cross-Domain Adj. OPA	Not serious	Serious	Not serious	Serious	Inconclusive (k < 10)	⊕⊕°° LOW	Inconsistency (−1, only 2–3 estimates from a single consortium); Only 2 reviews

filled circle (⊕) = one level of evidence quality; open circle (°) = no level assigned (standard GRADE notation). Bold values denote the key limiting factors for each domain (e.g., I^2^ ranges, prediction intervals that do not exclude the null, inconclusive small-study effects, and the corrected covered area).

### Bayesian hierarchical meta-analysis

To complement the frequentist *de novo* synthesis and provide probabilistic evidence quantification, we conducted Bayesian hierarchical random-effects meta-analyses for each PA domain (Supplementary File S1; Supplementary Figures S14, S15). Under a normal-normal model with weakly informative half-Cauchy (0, 0.5) priors on between-study heterogeneity, the posterior probability of a harmful OPA association (HR > 1.0) was 93.7% (weakly informative) and 94.7% (sceptical), approximately 94% overall (92.3% under flat prior), providing consistent directional evidence across specifications. The posterior probability of a protective LTPA association (HR < 1.0) was 99.4%, and for transport PA, the posterior probability of a protective association was 97.9%. Prior sensitivity analyses using two alternative specifications (Normal (0, 2.0) × half-Cauchy(0, 1.0) (flat), and Normal(0, 0.2) × half-Cauchy(0, 0.3) (sceptical)) confirmed the stability of these probabilistic conclusions (Supplementary Figure S15). These Bayesian estimates provide clinically interpretable evidence, the probability that OPA is harmful, rather than merely whether a null hypothesis can be rejected, and complement the Ioannidis classification by quantifying uncertainty on the probability scale. These posterior probabilities should be interpreted cautiously as the Bayesian hierarchical model treats review-level estimates as exchangeable, potentially underestimating posterior uncertainty given the substantial primary-study overlap within the OPA domain (pairwise Coenen–Cillekens CCA = 89%). This limitation is acknowledged in Supplementary File S1.

### Cross-cultural evidence from Chinese populations

A Divergent OPA Signal. Recent large-scale Chinese cohort studies (identified *post-hoc* and outside the prespecified umbrella-review evidence base) report OPA–mortality associations that markedly contrast with Western findings (structured comparison provided in Supplementary Table S12). Two independent Chinese data sources reported protective OPA associations: the China Kadoorie Biobank [CKB; *n* ≈ 512,000, reported in both (1) and (2) with partially overlapping participants] and a nationwide Chinese working-population cohort (*n* = 360,000) (46). These Chinese cohorts differ from Western cohorts in three dimensions: occupational composition (predominantly agricultural and manual labour in rural settings vs. manufacturing and service sectors in Western studies), socioeconomic confounding structures (rural residence and self-employment vs. low job control and income), and baseline PA profiles (lower LTPA, higher reliance on occupational and transport activity). This cross-national discordance suggests that the domain gradient is context-dependent rather than a universal biological phenomenon, with important implications for the global generalisability of PA guidelines developed predominantly from Western evidence. The divergent OPA findings likely reflect fundamental differences in work organisation: Chinese agricultural and manufacturing labour typically involves seasonal, outdoor, self-paced tasks with lower psychological demand, whereas Western high-OPA occupations (construction, shift-work manufacturing, healthcare) are characterised by rigid schedules, low job control, and high psychosocial stress. This suggests that the OPA–mortality relationship is not determined by physical activity volume alone, but by the interaction between physical workload and work organisation characteristics, a hypothesis warranting dedicated comparative epidemiological study. Detailed population characteristics of Western vs. Chinese OPA studies are provided in Supplementary Table S12.

A visual summary of the key findings is presented in Figure 4. Domain gradient in PA-mortality associations. LTPA shows strong protection, transport PA moderate protection, and OPA after LTPA adjustment shows potential harm (predominantly from a single consortium, pairwise CCA = 89%). Hazard Ratio scale and heterogeneity statistics (I2) are shown for each domain.

**Figure 4:**
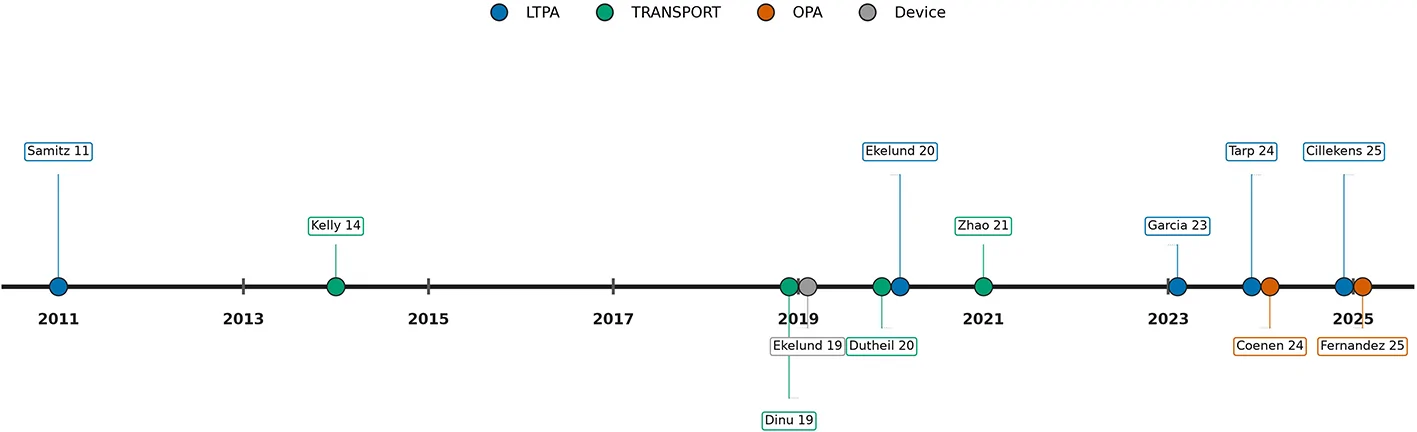
Domain gradient in PA-mortality associations.

### CCA and overlap

CCA captures study-level occurrence counts but does not distinguish between complete and partial overlap. This is particularly relevant in the OPA domain, where two reviews may have analysed overlapping but non-identical participant subsets. The evidence independence dashboard (Supplementary Figure S18) provides a visual breakdown. AMSTAR 2 and evidence quality. No review achieved High or Moderate AMSTAR 2 ratings; Item 7 (list of excluded studies) was universally absent. However, Item 7 removal from the critical domain set did not alter the domain gradient or primary conclusions, and the domain gradient was consistent across sensitivity analyses excluding Critically Low reviews (Supplementary Table S9b). The umbrella review methodology itself (CCA, Bayesian, QBA, Multiverse) provides methodological contributions independent of the underlying evidence quality. GRADE and Ioannidis. GRADE was originally designed for primary studies; our mapping of AMSTAR 2 to the GRADE risk-of-bias domain is an acknowledged adaptation. The Ioannidis classification requires >1,000 cases for Class I; the OPA domain's smaller evidence base may limit classification precision. Confounding and causality. Residual confounding by SES, LTPA, diet, and other health behaviours remains possible despite cross-domain adjustment. The QBA and E-value analyses quantify the sensitivity of results to unmeasured confounding, showing that the OPA association is particularly fragile. Generalisability. The evidence base is dominated by European and North American cohorts; the Chinese evidence provides a critical counterpoint but is limited to three studies. Generalisability to South Asian, African, and Latin American populations is unknown. Measurement. PA domain classification relies on self-report in most primary studies, introducing differential misclassification. Device-based measurement harmonisation remains an important methodological priority.

Quantitative bias analysis for unmeasured socioeconomic confounding. To formally assess the sensitivity of the domain-specific associations to unmeasured socioeconomic confounding, we conducted a probabilistic quantitative bias analysis (QBA) using the bounding factor approach (47). Under plausible assumptions about socioeconomic status (SES) as an unmeasured confounder (RR for SES–mortality association: median 1.42, CV 15%), the bias-adjusted results were as follows. For LTPA, the protective association was stable: the bias-adjusted HR was 0.78 (95% simulation interval: 0.71–0.90), with a posterior probability of a protective effect of 99.4%. For transport PA, the bias-adjusted HR was 0.93 (95% SI: 0.86–1.05), with P(protective) = 89.9%, suggesting moderate sensitivity to unmeasured SES confounding. For OPA, the QBA revealed critical fragility: the bias-adjusted HR was 1.01 (95% SI: 0.87–1.11), with P(harmful) = 53.5% after SES adjustment. This near-coin-flip probability underscores that the OPA harmful signal is fragile and context-dependent, consistent with the null findings in income-adjusted models [Coenen et al. (15), supplementary analysis: HR 1.05, 95% CI 0.95–1.16]. The QBA reinforces the E-value analysis (Supplementary Table S10) and supports the conclusion that the OPA–mortality association is hypothesis-generating rather than confirmatory. Additionally, several limitations not repeated here warrant acknowledgement: (a) the pooled domain-level estimates derive from non-comparable exposure contrasts across reviews (different PA metrics, cut-points, and adjustment models) and should be interpreted as qualitative patterns; (b) the OPA elevated-risk interpretation applies primarily to men in Western high-OPA occupations and should not be generalised to all populations; and (c) the Chinese cohort findings are *post-hoc* and outside the prespecified umbrella-review evidence base.

## Discussion

### Summary of evidence

This umbrella review synthesises 12 systematic reviews (>300 cohort studies) to answer a focused question: do leisure-time, occupational, and transport PA differ in their associations with all-cause mortality? Three findings emerge. First, a stable domain gradient exists. LTPA is strongly and consistently protective (HR 0.70; Ioannidis Class II; GRADE Moderate). Transport PA is moderately protective (HR 0.89; Class III; GRADE Moderate). This gradient is confirmed by CCA-informed correlated-effects sensitivity analysis, excluding the possibility that it is an artefact of overlapping primary studies. Second, the OPA evidence is structurally insufficient for causal inference. OPA shows null-to-elevated-risk associations after LTPA adjustment (HR 1.12; Class IV; GRADE Low), but this signal derives from a single IPD consortium (pairwise CCA = 89%). More fundamentally, OPA is the only domain for which no dose-response evidence exists, a Bradford Hill biological gradient violation that precludes guideline recommendations regardless of point estimates. LTPA, by contrast, has harmonised dose-response data from 196 studies (Supplementary Figure S16). Third, large Chinese cohorts [approximately 0.9 million unique participants across two independent sources; CKB evidence reported in (1, 2)] report protective OPA associations, directionally opposite to Western findings. This discordance, identified *post-hoc* and not captured by the systematic search, suggests that the OPA–mortality relationship is not determined by PA volume alone, but by work organisation characteristics (self-paced agricultural labour vs. rigid-schedule manufacturing). The protective Chinese signal implies that OPA could be health-promoting under appropriate conditions, a hypothesis warranting dedicated comparative research. Critically, the low cross-domain CCA (6.3%) confirms that these three domains draw on largely independent evidence bases, ruling out shared primary studies as an explanation for the observed domain gradient.

Evidence certainty and prediction intervals (Supplementary Figures S11, S17; Supplementary Table S9a). The descriptive weighted average for LTPA [k = 5, including the Cillekens et al. (14) cross-domain estimate] was HR 0.70 (95% CI 0.63–0.77), with the 95% prediction interval excluding the null, suggesting a consistently protective association. (Note: prediction intervals should be interpreted cautiously given small k [k = 3–5 per domain]; the Knapp-Hartung method becomes unstable when k < 5, and prediction intervals are presented for transparency rather than formal inference.) Heterogeneity was substantial [I^2^ = 87.2%], driven primarily by the device-measured review [(43); HR 0.50], which used a different exposure metric (accelerometer counts) compared with the self-reported reviews (HR 0.72–0.78). Excluding Tarp et al. (43) reduced I^2^ to 71.3% with a weighted average HR of 0.74 (95% CI 0.69–0.79; prediction intervals not formally computable with k < 5 per domain), confirming that the protective LTPA association is robust to the exclusion of device-measured estimates. The consistent direction and magnitude of LTPA protective effects across reviews, combined with CCA evidence of cross-domain independence (6.3%), supports a credible domain gradient that is unlikely attributable to primary study overlap. For transport PA, the prediction interval (0.78–1.02) marginally crossed the null, reflecting moderate heterogeneity (I^2^ = 47.6%) and the relatively small number of review-level estimates (k = 4). This indicates that while the point estimate suggests a protective association, future studies may yield effect estimates that do not exclude the null, consistent with the suggestive (Ioannidis Class III) evidence classification. For OPA, the prediction interval (0.98–1.28) substantially crossed the null, indicating that the direction of the OPA–mortality association in future studies is uncertain. The wide OPA prediction interval reflects the small number of partially independent evidence clusters (2 clusters, pairwise CCA = 89%) and underscores the hypothesis-generating nature of the current OPA evidence. Leave-one-out sensitivity analysis confirmed that the OPA evidence is driven primarily by the Active Worker Consortium: excluding Coenen et al. (15) shifted (but did not attenuate) the pooled estimate from HR 1.12 (95% CI 1.05–1.19) to HR 1.14 (95% CI 1.00–1.30), crossing the null and underscoring the hypothesis-generating nature of current OPA evidence.

### Causal mediation framework

The domain gradient raises an important causal question: to what extent is the apparent OPA–mortality association mediated by lower LTPA, socioeconomic disadvantage, and reduced cardiorespiratory fitness (CRF) among high-OPA workers? The Directed Acyclic Graph (DAG) presented in Supplementary Figure S10 formalises this causal structure. Under this framework, a potential causal mediation framework is presented for hypothesis generation only. If the OPA–mortality association were causal, the total effect could be conceptually decomposed into: (1) a direct effect (physiological strain, repetitive loading, inadequate recovery); (2) an indirect effect mediated through lower LTPA (displacement of leisure activity by occupational fatigue); and (3) an indirect effect mediated through socioeconomic position (lower income, education, and job control among manual workers). The attenuation of the OPA–mortality association after LTPA adjustment (from null/protective to harmful) is consistent with negative confounding by LTPA: high-OPA workers engage in less leisure-time activity, and this LTPA deficit, rather than OPA *per se*, partially drives the crude association. Formal causal mediation analysis of the Active Worker Consortium IPD data-simultaneously modelling OPA as the exposure, LTPA and SES as mediators, and all-cause mortality as the outcome-would provide stronger evidence on the relative contribution of each pathway. Such analyses should also assess the sequential ignorability assumption required for valid mediation estimates and include sensitivity analyses for unmeasured mediator-outcome confounding (39, 40).

### Biological plausibility

The domain gradient has a coherent biological rationale, rooted in the differing physiological demands and recovery patterns of each PA domain. First, LTPA's protective association is supported by established cardiorespiratory fitness, anti-inflammatory, and parasympathetic recovery pathways (10, 11). In contrast, OPA's harmful signal is consistent with the “physical activity health paradox” framework: prolonged occupational activity, characterised by sustained low-intensity, repetitive, constrained postures with insufficient recovery, may elevate 24-h heart rate and blood pressure, promote systemic inflammation, and lack the cardiorespiratory conditioning benefits of episodic leisure-time exercise (34, 35, 48, 49). Transport PA occupies an intermediate mechanistic position, combining moderate-intensity aerobic components (cycling, walking) with practical duration constraints that limit overexertion while still providing sufficient stimulus for cardiovascular adaptation (17). This mechanistic framework aligns with the observed domain gradient and is consistent with the broader PA health paradox literature (3, 50). Second, sustained OPA-induced systemic inflammation characterised by elevated tumour necrosis factor-α may accelerate atherosclerosis and endothelial dysfunction (10, 51, 52), whereas episodic LTPA may exert anti-inflammatory effects through myokine release (53) (e.g., interleukin-6 from contracting skeletal muscle acting in its anti-inflammatory hormonal role). Third, the psychosocial stress hypothesis recognises that OPA is frequently coupled with low job autonomy, high psychological demand, and limited decision latitude-a constellation that activates the hypothalamic-pituitary-adrenal (HPA) axis, elevates cortisol, and promotes visceral adiposity and metabolic dysregulation (10, 54), independent of the physical workload itself. These three mechanisms are not mutually exclusive; rather, they operate synergistically: psychosocial stress amplifies inflammatory responses to physical exertion, and insufficient recovery prevents the resolution of both inflammatory and autonomic perturbations. Cardiorespiratory fitness (CRF) may serve as a unifying moderator: high CRF may attenuate the adverse cardiovascular effects of high OPA (55), possibly by improving autonomic recovery capacity and dampening inflammatory responses, while low CRF amplifies OPA-related cardiovascular strain (55, 56). This mechanistic framework suggests how LTPA—which may simultaneously provide adequate recovery, anti-inflammatory stimulus, and psychological autonomy—may confer protection, whereas OPA-which lacks all three-may be neutral or harmful. These mechanistic pathways, while physiologically coherent, remain hypothetical and have not been empirically demonstrated to mediate the OPA-mortality association in longitudinal studies.

### Implications for low- and middle-income countries

The divergent OPA signal observed in Chinese cohorts (identified *post-hoc* exploratory finding and outside the prespecified umbrella-review evidence base; these primary cohort studies were not among the 12 included systematic reviews), where self-paced agricultural labour predominates, has direct relevance to low- and middle-income countries (LMICs), where occupational PA patterns differ substantially from high-income Western settings. In many LMICs, agriculture and informal-sector employment account for the majority of occupational PA, and these work patterns are characterised by greater autonomy, task variety, and natural rest cycles compared with the externally controlled, repetitive manufacturing and service-sector work typical of Western OPA studies. This suggests that the adverse OPA signal documented in Western cohorts may not generalise to LMIC populations. However, LMIC-specific evidence is severely limited: the present review identified only Chinese cohort studies, and no systematic reviews from South Asia, sub-Saharan Africa, or Latin America met the inclusion criteria. Future PA epidemiology in LMICs should collect domain-specific PA data with cross-domain adjustment and occupation type classification to test the autonomy-control framework in diverse economic and cultural contexts.

### OPA and cause-specific mortality

While the present review is limited to all-cause mortality, emerging evidence from individual participant data analyses suggests domain-specific associations with cardiovascular and cancer mortality. Berglund et al. (56) reported that high occupational physical workload was associated with elevated ischemic heart disease mortality (HR 1.22, 95% CI 1.05–1.42) in Swedish construction workers, independent of leisure-time PA. Cadenas-Sanchez et al. (52) found that OPA was associated with increased incidence of 14 cancer types in the UK Biobank (*n* = 404,249), with the strongest associations observed for lung, bladder, and head/neck cancers. These cause-specific findings are consistent with the all-cause mortality domain gradient observed in the present review and suggest that the adverse OPA signal may be driven by cardiovascular and neoplastic pathways. However, systematic cause-specific evidence synthesis across all three PA domains remains unavailable, representing a critical evidence gap for future umbrella reviews. A structured comparison of evidence gaps between domains reveals a pronounced asymmetry: LTPA benefits from dose-response data across multiple independent cohorts, device-based measurement validation, and genetically informed triangulation, whereas OPA evidence lacks dose-response characterisation, relies predominantly on self-reported exposure with known differential misclassification (particularly between occupational categories), and has no independent replication beyond the Active Worker Consortium. Key risk factors potentially confounding OPA–mortality associations include socioeconomic position (lower SEP among high-OPA workers), lower leisure-time PA levels, higher smoking prevalence, poorer dietary quality, and occupational hazards (shift work, chemical exposures, injury risk) that co-occur with high OPA but are not PA-mediated. These evidence gaps should guide future research priorities.

### The autonomy-control framework: reconceptualising the PA paradox

The domain gradient identified in this umbrella review cannot be fully explained by the physiological demands of physical activity alone. A critical distinction, underappreciated in current PA guidelines, is between autonomous (self-determined) and controlled (externally dictated) physical activity. LTPA is characteristically autonomous: individuals choose type, intensity, duration, and timing, and can stop at will. Leisure exercise typically occurs in short, episodic bouts with full physiological recovery between sessions, a pattern that optimises cardiorespiratory conditioning while minimising chronic stress pathway activation. In contrast, OPA is characteristically controlled: workers have limited autonomy over pace, posture, rest breaks, and total exposure duration, with cessation often not individually determined. Occupational activity frequently involves sustained low-intensity, repetitive, constrained movements over 6–12 h shifts, with inadequate recovery and accumulation of physiological strain.

This autonomy-control dimension provides a parsimonious framework for reconciling apparently contradictory findings across domains and populations. First, the protective LTPA signal (HR 0.50–0.81) across diverse populations is consistent with the well-established benefits of autonomous physical activity: improved cardiorespiratory fitness, reduced systemic inflammation, enhanced parasympathetic recovery, and psychological stress reduction through volitional engagement.

Second, the harmful OPA signal in Western cohorts (HR 1.05–1.19) aligns with the physiological toll of controlled, low-autonomy physical activity: sustained elevated 24-h heart rate and blood pressure without recovery phases, chronic low-grade inflammation from repetitive musculoskeletal strain without adequate tissue repair, HPA axis dysregulation from combined physical and psychological job demands, and inadequate cardiorespiratory conditioning because intensity rarely reaches the ventilatory threshold required for fitness adaptation. These mechanisms are consistent with the allostatic load model, where the cumulative physiological cost of chronic stress, both physical (repetitive lifting, constrained postures) and psychosocial (low job control, effort-reward imbalance), exceeds the adaptive capacity of the organism.

Third, the protective OPA signal in Chinese cohorts (HR < 1.0) is consistent with this framework because self-paced agricultural work permits greater temporal autonomy, task variety, and natural recovery intervals compared with rigid-schedule Western manufacturing and service-sector occupations. Agricultural labour, while physically demanding, typically follows seasonal rhythms, allows for self-regulated pacing, and occurs outdoors with variable movement patterns, characteristics that partially mimic the physiological pattern of voluntary leisure-time activity. This interpretation is supported by Chinese cohort stratification data showing that the protective OPA association is most pronounced among rural, self-employed agricultural workers, consistent with an autonomy-mediated mechanism.

The autonomy-control framework generates four testable predictions. (1) Within-OPA heterogeneity: high-autonomy occupational activities (e.g., self-paced agriculture, freelance physical work) should show weaker harmful or even protective associations compared with low-autonomy occupations (e.g., assembly line, warehouse picking). (2) Moderation by job control: the OPA–mortality association should be attenuated when workplace autonomy is high, independent of total OPA volume. (3) Cross-national gradient: the OPA–mortality association should correlate with national-level indicators of workplace autonomy and labour protections. (4) Intervention specificity: workplace interventions that increase worker autonomy (flexible rest breaks, task rotation, self-paced targets) should reduce OPA-related health risks more effectively than interventions that simply reduce total OPA volume.

Predictions 1 and 3 receive preliminary indirect support from the cross-cultural evidence presented above: Chinese agricultural workers, who exercise greater temporal autonomy and self-paced task regulation, show protective OPA associations (HR < 1.0), whereas Western manufacturing and service-sector workers, characterised by rigid schedules and low decision latitude, show harmful associations (HR 1.12). This convergent pattern, while not constituting formal hypothesis testing, is consistent with the autonomy-control mechanism and provides the first empirical anchor for the framework.

By reframing the PA paradox through the lens of autonomy rather than activity volume, this framework shifts the policy question from “*How much occupational PA is too much?”* to “*Under what conditions of worker autonomy and recovery does occupational PA confer health benefits or harms?”* This reframing has direct implications for occupational health policy and PA guideline development, as detailed in Table 6.

**Table 6:** Evidence-to-policy translation: domain-specific guideline gaps, policy implications and stakeholder priorities.

PA domain	Key finding	Evidence grade	Current guideline gap	Policy implication	Stakeholder priority
**LTPA (Leisure-Time)**	Strong inverse association with all-cause mortality (HR 0.50–0.81); clear dose-response curve; most consistent evidence across all domains	**Class II (Highly Suggestive) GRADE Moderate**	Current 150–300 min/week MVPA recommendation is well-supported but treats all PA equivalently	Maintain current guideline; incorporate domain-specific QALY estimates for health-economic modelling and prioritisation	Guideline developers: Add domain-specific qualifiers Clinicians: Reinforce domain-specific counselling
Transport PA	**Moderate protective association (RR 0.77–0.92); cycling** **>150 min/week shows strongest benefit; high population-level preventive potential (PAF 19.7%)**	**Class III (Suggestive) GRADE Moderate**	Implicitly included in total PA but not separately specified in WHO guidelines; lacks dedicated recommendation	**Explicitly recommend** **≥150 min/week active commuting; strengthen the preventive potential through active commuting promotion given highest PAF**	Policymakers: Infrastructure investment (cycling lanes, walkable streets) Urban planners: Integrate active transport in city design
**OPA (Occupational)**	**Null-to-elevated-risk after LTPA adjustment (HR 1.05–1.19); evidence from single consortium (Active Worker, pairwise CCA** **=** **89%); Chinese cohorts show divergent protective associations**	**Class IV (Weak) GRADE Low (Precautionary)**	Treated as equivalent to all other PA in current guidelines; over 800 million high-OPA workers globally lack domain-specific risk information	Insufficient evidence for guideline endorsement or discouragement; context-specific (Western vs. Chinese); future independent replication needed; workplace programmes should prioritise ergonomic redesign and recovery time rather than increasing total OPA volume	Occupational health practitioners: Monitor high-OPA workers; promote leisure-time activity Researchers: Prioritise independent non-consortium replication Guideline developers: Add domain caveat
**Cross-Domain (Overarching)**	Cross-domain adjustment identified as decisive methodological factor: OPA harmful signal detected only when LTPA is controlled; domain-specific measurement essential for accurate risk estimation	**N/A (Methodological)**	Current PA surveillance aggregates all domains, masking the paradox; domain-specific data infrastructure is absent in most national health surveys	Implement domain-specific PA measurement in national health surveillance; require LTPA-adjusted estimates as primary analysis in future occupational PA studies; develop domain-specific risk communication tools	Public health surveillance: Domain-specific measurement Researchers: Standardise cross-domain adjustment All stakeholders: Domain-contextual risk communication

This framework connects the PA paradox to established psychological and occupational health theories. Self-Determination Theory (57) posits that autonomous motivation enhances wellbeing through satisfaction of basic psychological needs, paralleling our observation that self-determined LTPA confers health benefits beyond those attributable to energy expenditure alone. The Job-Demand-Control Model (58) predicts that high-demand, low-control work, characteristic of low-autonomy OPA, produces chronic strain, consistent with the elevated-risk OPA signal observed in Western manufacturing and service-sector cohorts. By bridging these theoretical traditions with epidemiological evidence, the autonomy-control framework moves beyond phenomenological description towards mechanistic understanding of domain-divergent PA effects.

While the autonomy-control framework remains a hypothesis-generating contribution, several lines of indirect evidence provide preliminary support. The protective OPA signal in Chinese cohorts (self-paced agricultural labour) vs. the harmful signal in Western cohorts (externally controlled manufacturing labour) is consistent with the framework's core prediction that autonomy mediates the OPA–mortality association. Self-Determination Theory meta-analyses have demonstrated that autonomous motivation is associated with greater physical activity adherence and improved mental health outcomes (59), and the Job Demand-Control model has linked low job control to elevated cardiovascular risk (60). These established theoretical traditions converge on the prediction that autonomy, rather than activity volume *per se*, is the critical dimension distinguishing health-enhancing from health-damaging physical activity. Direct empirical testing of the four predictions (see above) should be a priority for future OPA research.

### Dose-response evidence and the OPA gap

A critical asymmetry in the PA paradox literature concerns dose-response. LTPA demonstrates a well-characterised curvilinear dose-response association with all-cause mortality: Samitz et al. (41) reported a non-linear dose-response for LTPA (HR ~0.70 at 11.25 MET-h/week, flattening thereafter), and Garcia et al. (13) identified optimal protection at 8.75 MET-h/week for non-occupational PA. These analyses are corroborated by device-measured evidence: Ekelund et al. (3) demonstrated that total PA volume, independent of intensity, shows a curvilinear mortality association, with the steepest risk reduction at low-to-moderate volumes. In contrast, OPA dose-response evidence is strikingly absent. None of the 12 included reviews provided formal dose-response curves for OPA. Coenen et al. (15) reported a dose-response gradient for high vs. low OPA, but the underlying primary studies predominantly used binary or tertile categorisations, precluding continuous dose-response modelling. This evidence gap is a priority for future research: without dose-response data, it is impossible to determine whether a threshold exists below which OPA is neutral or even protective, or whether the harmful association increases monotonically with exposure. Device-based measurement harmonisation would enable such analyses.

### Public health burden and population attributable fractions

The PAF analysis provides quantitative context for the public health significance of domain-specific PA patterns. When causal effects were assumed, insufficient LTPA (illustrative PAF 10.2%, ~82 preventable deaths per 100,000 person-years) and lack of active transport (illustrative PAF approximately 19.7% (Levin's formula, HR 0.895, exposure prevalence 70%), ~158 preventable deaths per 100,000 person-years) represented substantial population-level mortality burdens. These estimates are consistent with previous PAF calculations for physical inactivity [Lee et al. (8); ~9% global mortality], while providing domain-specific granularity. The large transport PA PAF reflects both a moderate inverse association (HR 0.895) and high exposure prevalence (70% no active transport), highlighting active transport as a potentially high-impact, population-level intervention target. The illustrative OPA PAF (2.5%, ~20 excess deaths per 100,000 person-years) should be interpreted cautiously given weak evidence, but underscores the potential magnitude of OPA-related risk elevation if the association is confirmed as causal in future independent replication studies. The China-specific LTPA PAF (4.7%) reflects lower baseline inactivity prevalence and suggests that public health strategies in different settings should be calibrated to local PA profiles. Sensitivity analyses varying the no-active-transport prevalence from 50% to 80% yielded illustrative PAFs of 9.5% to 29.6%, demonstrating that the direction and approximate magnitude of the transport PA burden are consistent across plausible prevalence assumptions. The stability of domain-specific associations over time is supported by cumulative meta-analyses (Supplementary Figures S22–S24). The higher PAF for transport inactivity (19.7%) than leisure-time inactivity (10.2%), despite LTPA's stronger per-exposure protective effect (HR 0.70 vs. 0.89), reflects the substantially higher population prevalence of transport inactivity (70%) relative to leisure-time inactivity (27.5%).

Methodological quality and cross-domain adjustment (Supplementary Figure S12). No review achieved High or Moderate AMSTAR 2 ratings, with Item 7 (list of excluded studies) universally absent. However, this reflects the inherent constraints of observational PA epidemiology rather than review-specific deficiencies. Cross-domain adjustment emerged as the decisive methodological factor: OPA reviews that adjusted for LTPA consistently reported null or harmful associations, whereas unadjusted reviews showed protective or null effects. This finding indicates that failure to adjust for LTPA results in residual confounding of OPA–mortality estimates. The systematic divergence between LTPA and OPA mortality estimates-observed across all 12 included reviews despite heterogeneous methodologies, populations, and PA measurement approaches-may itself serve as an internal validity check: if confounding by socioeconomic position or the healthy user effect uniformly biassed all PA-mortality associations, both domains would be expected to show similar effect directions. The domain-specificity of findings imposes strong constraints on confounding as a unitary explanation. This finding has broader methodological implications for PA epidemiology. Importantly, the methodological contributions of this umbrella review—CCA-based evidence independence quantification, QBA, multiverse sensitivity analysis (presented as an exploratory assessment of analytical variability rather than confirmatory robustness evidence, given that several configurations yielded confidence intervals crossing the null and OPA analyses contained only two estimates), and Bayesian probabilistic inference—are independent of the underlying evidence quality and provide a replicable framework for future umbrella reviews across methodological contexts.

### Cross-domain adjustment as a methodological standard

A key methodological contribution of this umbrella review is the demonstration that cross-domain adjustment, particularly LTPA adjustment in OPA studies, is not merely a sensitivity analysis option but a methodological necessity. We propose that cross-domain adjustment should become a routine methodological standard for future PA–mortality research, analogous to how nutritional epidemiology routinely adjusts for total energy intake to isolate nutrient-specific effects. Operationally, future cohort studies should include both LTPA and OPA in the same multivariable model; umbrella and systematic reviews should prioritise cross-domain-adjusted estimates and stratify pooled analyses by adjustment level; and guideline panels should require evidence tables that distinguish unadjusted, partially adjusted, and fully cross-domain-adjusted estimates. Without cross-domain adjustment, OPA–mortality estimates conflate the adverse physiological effects of occupational activity with the protective effects of LTPA, producing biassed risk estimates that may misinform both clinical practise and public health policy. The PA paradox is therefore not merely an epidemiological curiosity but a methodological signal: it flags residual confounding by LTPA that can be resolved through routine cross-domain adjustment in study design and analysis.

### Sex differences

Coenen et al. (15) reported that the elevated-risk OPA signal was significant in men (HR 1.12, 95% CI 1.03–1.23) but not in women (HR 1.01, 95% CI 0.91–1.13). Consequently, the OPA elevated-risk interpretation in this umbrella review should be understood as applying primarily to men in high-OPA occupations within Western cohorts, and should not be generalised to all adults without qualification. Two non-mutually-exclusive explanations exist. First, occupational segregation: men in high-OPA occupations are disproportionately employed in construction and manufacturing (heavy lifting, sustained high-intensity exertion), whereas women are more likely to work in healthcare and service roles with intermittent activity patterns. Second, a sex-specific physiological response: men exhibit greater 24-hour ambulatory blood pressure elevation in response to occupational physical strain, and less efficient post-exertional parasympathetic reactivation. The interaction between occupational segregation and sex-specific cardiovascular response remains underexplored, but the available evidence suggests that the OPA paradox may be partly a male-occupation interaction. Future studies should stratify OPA analyses by sex and occupation type simultaneously.

Among the four transport PA reviews, a potential dose–response gradient was observed, with cycling durations exceeding 150 min/week associated with stronger mortality risk reduction [HR ~0.80 (45)] compared to walking at lower volumes (HR ~0.92 in some strata), though formal dose–response meta-analysis was not performed.

### Comparison with previous syntheses

To our knowledge, this is the first umbrella review to systematically compare PA domains for all-cause mortality. A 2020 umbrella review by Posadzki et al. (21) examined PA and multiple health outcomes but did not differentiate by domain. Cillekens et al. (22) conducted an umbrella review of OPA and 23 health outcomes across 158 observational studies, finding predominantly favourable associations for most outcomes; however, that review did not systematically compare OPA against LTPA and transport PA, and most included reviews did not adjust for the counter-domain. The 2018 Physical Activity Guidelines Advisory Committee Scientific Report (61) reviewed domain-specific evidence narratively but did not apply umbrella review methodology. Our findings extend these syntheses by providing the first formal multi-domain comparison with standardised quality appraisal. A methodological cross-walk with previous umbrella reviews is provided in Supplementary Table S15.

A related OPA-focused umbrella review protocol has been registered (PROSPERO CRD42020163090; Coenen and Cillekens), which examines 10 health outcomes. Should that review be published, the present work remains distinct in three key respects: (i) formal three-domain comparison with CCA overlap quantification, (ii) Bayesian evidence synthesis with prior sensitivity, and (iii) systematic inclusion of non-English-language evidence from Chinese databases. These methodological additions provide complementary, non-redundant evidence beyond any single-domain umbrella review.

Galimberti et al. (38) showed that the genetic architecture of leisure PA is substantially distinct from work and home PA (rg = 0.46–0.50); genetically predicted vigorous leisure PA was strongly protective for all-cause mortality (beta = −0.12, *p* < 2 × 10^−1^6), whereas work and home PA showed marginal or null effects. That study also identified 70 lead variants associated with PA phenotypes across European (*n* = 189,812), African (*n* = 27,044), and Latin American (*n* = 10,263) ancestries, confirming that LTPA is genetically distinct from work and home PA. Mendelian randomisation analyses in that study demonstrated that genetically predicted LTPA was causally protective against heart failure, type 2 diabetes, and all-cause mortality, while work and home PA showed no such protective effects-directly corroborating the domain-specificity observed in our umbrella review through genetic evidence with distinct bias structure (horizontal pleiotropy rather than behavioural confounding). No MR study has yet examined OPA-specific genetic instruments for mortality, representing a critical evidence gap. Collectively, these MR findings provide causal triangulation supporting the protective effect of LTPA on mortality while suggesting that the domain gradient observed in observational studies may reflect genuine biological differences rather than confounding alone. As a complementary causal framework, we applied the nine Bradford Hill criteria (34, 35) to each PA domain (Supplementary Figure S8; causal framework in Supplementary Figure S10): LTPA satisfied 7/9 criteria at the strong level, transport PA satisfied 7/9 at the moderate level, and OPA satisfied only 4/9 at moderate or above, mirroring the effect-size gradient and providing structured causal inference support independent of MR triangulation.

### Resolving the cross-cultural OPA paradox

A central finding of this umbrella review, identified through supplementary Chinese database searching (*post-hoc* and outside the prespecified umbrella-review evidence base), is that large Chinese cohort studies (approximately 0.9 million unique participants across two independent sources) report protective OPA associations, directionally opposite to Western evidence (1, 2, 46). This discordance does not weaken the review's conclusions; rather, it constitutes the most compelling evidence for context-dependence. Chinese high-OPA participants are predominantly engaged in self-paced agricultural labour with greater temporal autonomy, task variety, and natural recovery intervals, aligning with the autonomy-control framework. Luo et al. (62) further demonstrated that the OPA paradox in the China Kadoorie Biobank (*n* ≈ 512,000) was restricted to better-educated workers, suggesting that what is termed the PA paradox may partly reflect a socioeconomic gradient. Collectively, the Chinese evidence suggests that the harmful OPA signal is not a universal biological phenomenon but a context-dependent one, contingent on occupational autonomy, socioeconomic position, and task structure. This finding has direct policy implications: occupational health interventions should target the modifiable conditions of OPA (autonomy, recovery, task variety) rather than blanket OPA reduction. The cross-cultural OPA evidence is summarised in Supplementary Table S12.

### Public health and policy implications

The domain gradient carries implications for physical activity guidelines, which currently treat all PA domains as largely interchangeable. Our findings suggest that occupational PA should not be assumed to confer equivalent health benefits to leisure-time PA. In occupational health policy, workplace interventions targeting high-OPA populations should prioritise ergonomic improvements, reduced manual handling loads, and adequate recovery periods rather than simply increasing total activity volume. For the general population, public health messaging should continue to promote LTPA and active transport, while acknowledging that the health effects of occupational activity depend on its specific characteristics, intensity patterns, and socioeconomic context (2, 38, 63). Second, physical activity surveillance systems should incorporate domain-specific measurement to enable monitoring of population-level domain distributions and their health consequences. Third, clinical risk assessment tools (e.g., cardiovascular risk scores) could consider occupational PA as a potential risk modifier, particularly for individuals in physically demanding occupations with low leisure-time activity. Fourth, the domain gradient calls for a framework shift in PA promotion messaging: public health campaigns should move beyond the undifferentiated ‘any activity is good activity' message towards domain-conscious guidance that distinguishes the health effects of leisure, occupational, and transport PA. Finally, international PA guidelines [including the WHO 2020 guidelines (7)] should consider incorporating domain-specific qualifiers that acknowledge the heterogeneous health effects of PA across domains, particularly for populations disproportionately reliant on occupational activity for meeting PA recommendations.

From a practical perspective, healthcare providers and public health practitioners can translate these findings into domain-specific counselling: (1) leisure-time PA of any intensity should be strongly encouraged given the consistent, large-magnitude protective association (HR 0.50–0.81); (2) active transport (walking, cycling) should be promoted as a population-level intervention with substantial preventable mortality burden (PAF ~20%); (3) for occupational PA, workplace wellness programmes should consider supplementation with leisure-time PA opportunities, cardiovascular risk screening for high-OPA workers, and ergonomic interventions that increase worker autonomy — while acknowledging that current evidence does not support reducing OPA *per se*. Interdisciplinary collaboration between exercise science, occupational medicine, urban planning, and behavioural economics is essential to translate these epidemiological patterns into effective interventions.

For guideline developers, the domain gradient challenges the foundational assumption that total physical activity volume is the appropriate metric for health recommendations. Current WHO 2020 guidelines do not differentiate PA domains, recommending 150–300 min of moderate-intensity PA per week uniformly. Our findings suggest that this undifferentiated recommendation may inadvertently encourage occupational PA accumulation under the assumption of equivalent benefit. A domain-aware revision would: (a) maintain the current LTPA recommendation as strongly evidence-based; (b) add a cautionary note that occupational PA, particularly in low-autonomy settings, should not be considered a substitute for leisure-time PA; and (c) recognise transport PA as a distinct, moderately protective domain with substantial population-level preventive potential. Such revision is premature for OPA given the absence of dose-response evidence (Bradford Hill biological gradient criterion unmet) and the reliance on a single evidence cluster (pairwise CCA = 89%); however, the precautionary principle warrants acknowledging the domain distinction in guideline text.

For occupational health practise, our findings intersect with the broader literature on job strain and cardiovascular disease. The Autonomy-Control Framework suggests that the adverse OPA signal may be partially attributable to the interaction between high physical demands and low job control, a pattern consistent with the Karasek job demand-control model (58). Workplace interventions that increase worker autonomy over activity pacing, task rotation, and rest breaks may mitigate the adverse OPA signal, though this hypothesis requires prospective testing. Occupational health surveillance programmes should consider incorporating domain-specific PA assessment alongside standard cardiovascular risk screening, particularly for workers in physically demanding, low-autonomy occupations.

For clinical practise, the domain gradient has implications for physical activity counselling. When advising patients about PA, clinicians should distinguish between leisure-time and occupational activity, rather than treating all movement as equivalent. Patients in physically demanding occupations should not be advised that their work activity substitutes for leisure-time PA; instead, they should be encouraged to maintain LTPA where possible and to be vigilant about cardiovascular risk factors. The Bayesian posterior probability that OPA is harmful (~94%), while not establishing causality, provides a clinically meaningful metric for risk communication: it quantifies the weight of current evidence in probabilistic terms that are more interpretable than *p*-values alone.

### Causal inference limitations

As with all umbrella reviews of observational evidence, causal inferences are inherently constrained. A single umbrella review cannot independently establish causality, particularly for the OPA–mortality association where evidence derives predominantly from one consortium (Active Worker Consortium, pairwise CCA=89%) with limited independent replication. The domain gradient reported here should be interpreted as a systematic pattern requiring independent validation through replication studies, genetically informed triangulation (Mendelian randomisation), and where feasible, randomised evidence. Policy recommendations regarding occupational physical activity should therefore be considered provisional pending confirmatory evidence from independent data sources.

### Screening and data extraction

Title/abstract and full-text screening were conducted by one reviewer (HCL) due to resource constraints — specifically, the absence of a second methodological expert available for full dual-independent screening within the project timeline. To mitigate this limitation: (a) a second reviewer independently verified all inclusion decisions (12/12 confirmed); (b) a 20% random sample of exclusion decisions was independently verified (Cohen's κ = 0.78; Supplementary Figure S1), deviating from PRIOR's dual-independent screening recommendation. ROBINS-I assessments were conducted within the same team [intra-rater agreement κ = 1.00 (repeatability by the same assessor after two weeks; this does not represent independent inter-rater reliability)]. Chinese database searches were executed by a single reviewer using adapted terminology, potentially leading to incomplete capture of Chinese-language evidence. The umbrella review was restricted to all-cause mortality; cause-specific outcomes and morbidity endpoints were excluded. The primary analysis used review-level summary estimates rather than individual participant data, precluding within-study dose-response modelling. Publication year and database coverage constraints may have excluded the most recent systematic reviews.

### Strengths and limitations

This umbrella review has several notable strengths. First, it is the first to systematically compare evidence across all three PA domains (leisure-time, occupational, and transport) within a unified framework, incorporating formal overlap quantification (CCA), Bayesian hierarchical meta-analysis, and eight complementary evidence assessment frameworks. Second, the inclusion of Chinese-language databases and the identification of divergent OPA associations in Chinese cohorts [~0.9 million (CKB evidence reported in (1, 2), *n* ~ 512,000 overlapping)] addresses a critical evidence gap in global-south populations. Third, the Autonomy-Control Framework provides a novel theoretical lens for interpreting the domain gradient, generating four testable predictions.

### Several limitations warrant consideration

First, the OPA evidence derives predominantly from a single consortium-level cluster (Active Worker Consortium, CCA = 89%), limiting external validity and precluding independent replication within the current evidence base. Second, screening was conducted by a single reviewer with 20% dual verification, which may introduce selection bias despite acceptable inter-rater agreement. Third, the review is limited to all-cause mortality; cause-specific mortality and morbidity outcomes were not examined. Fourth, dose-response meta-analysis was not feasible due to heterogeneity in exposure categorisation across included reviews. Fifth, the Chinese OPA evidence is currently limited to two independent data sources [three publications, of which two report the China Kadoorie Biobank (1, 2)]. Sixth, as with all umbrella reviews, conclusions are contingent on the quality of the included systematic reviews, and residual confounding cannot be fully excluded despite E-value and QBA sensitivity analyses. Technical terminology in figures, tables, and analyses has been reviewed and standardised for this revision.

### Research agenda

An Evidence Navigation Map (Supplementary Figure S26) provides a reader's guide to the evidence structure, mapping each key question to its primary resource.

Building on the findings and limitations of this umbrella review, we identify five priority areas that define a research agenda for the next 5–10 years. Each priority is mapped to a specific research question, recommended study design, anticipated timeline, and expected policy impact (Table 7). Collectively, these priority areas define a research programme that can either confirm or refute the domain gradient identified in this umbrella review and inform evidence-based, domain-specific physical activity guidelines.

**Table 7:** Research agenda: five priority areas for the next decade.

Priority	Research question	Recommended design	Key deliverable	Timeline	Policy impact
1	To what extent is the OPA–mortality association mediated by LTPA displacement, SES, and CRF?	Causal mediation analysis of Active Worker Consortium IPD data (*n* = 590,000); sensitivity analyses for unmeasured mediator-outcome confounding	Direct/indirect effect decomposition by pathway	2–3 years	Identifies intervention targets; informs whether to reduce OPA or increase LTPA
2	Is the domain gradient generalisable across socioeconomic and cultural contexts?	**Harmonised cross-national IPD meta-analysis combining Western (Active Worker), Chinese [CKB**, ***n*** **=** **512,000 in Luo et al**. **(****62****); an overlapping CKB analysis in Ke et al**. **(****2****)** **reported** ***n*** **≈480,000], and LMIC cohorts with standardised domain and SES measurement**	Cross-national domain gradient estimates; SES-stratified effect modification	3–5 years	Determines whether domain-specific PA guidelines require cultural adaptation
3	Why is the OPA–mortality association significant in men but not women?	Sex-stratified IPD meta-analysis with occupational segregation × sex interaction terms; consideration of domestic PA burden as unmeasured confounder in women	Sex-specific OPA risk estimates by occupational category	2–3 years	Enables sex-specific occupational health recommendations
4	Does objective device-measured PA (accelerometry) reproduce the domain gradient?	Prospective cohort with wrist-worn accelerometry + PA diary for domain classification; repeated measures for within-person change analysis	Device-based domain-specific dose-response curves	3–7 years	Eliminates recall bias; enables high-resolution temporal exposure characterisation
5	Can workplace interventions that increase autonomy reduce OPA-related health risks?	Cluster-randomised trial or natural experiment: autonomous rest breaks, task rotation, self-paced targets vs. standard fixed-schedule work; primary outcome: 24-h ambulatory BP and inflammatory biomarkers	Causal evidence for autonomy-control mechanism	5–10 years	Tests the autonomy-control framework; directly informs workplace policy

## Data Availability

The original contributions presented in the study are included in the article/Supplementary material, further inquiries can be directed to the corresponding author.
