# Supplementary material for: The physical activity paradox and all-cause mortality: an umbrella review of systematic reviews and meta-analyses
Source: Front Public Health. 2026 Oct 28;14:1934173. doi: 10.3389/fpubh.2026.1934173 (PMC13751947; doi:10.3389/fpubh.2026.1934173)
Supplement: Supplementary file 1 [file Supplementary_file_1.docx]

# Supplementary Materials

The Physical Activity Paradox and All-Cause Mortality: An Umbrella Review of Systematic Reviews and Meta-Analyses

Lin HC, Zhao H. Guilin College, School of Physical Education and Health.

PROSPERO: CRD42026142431

**Guide to Supplementary Materials**

This document contains all supplementary materials. Contents are organised as follows:
Supplementary Tables
Table S1
Table S1b
Table S1c
Table S1d
Table S1e
Table S1f
Table S1g
Table S1h
Table S1i
Table S2a
Table S2b
Table S3
Table S3a
Table S4
Table S8
Table S8a
Table S9a
Table S9b
Table S10
Table S11
Table S12
Table S12a
Table S14
Table S15
Table S16
Table S17
Supplementary Figures
Figure S1
Figure S2
Figure S3
Figure S4
Figure S5
Figure S6
Figure S7
Figure S8
Figure S9
Figure S10
Figure S11
Figure S12
Figure S13
Figure S14
Figure S15
Figure S16
Figure S17
Figure S18
Figure S19
Figure S20
Figure S21
Figure S22
Figure S23
Figure S24
Figure S25
Figure S26
Supplementary File S1: Interactive Evidence Dashboard
Supplementary Text S1: Bayesian Hierarchical Meta-Analysis

Table S1: Complete Search Strategy, PRISMA Flow Diagram, and Exclusion Reasons

Table S2a: Corrected Covered Area (CCA) Analysis by PA Domain

Table S2b: Pairwise CCA Matrix Within Domains

Table S3: AMSTAR 2 Item-Level Scoring for All 12 Included Reviews

Table S3a: CCA-Informed Descriptive Synthesis — Forest Plot Data

Table S4: P-Curve Analysis and Excess Significance Testing

Table S8: ROBINS-I Risk of Bias Assessment — Active Worker Consortium Cohorts

Table S8a: Exposure Definitions and Adjustment Strategies Across Included Reviews

Table S9a: CCA-Informed Descriptive Synthesis Results and Sensitivity Analysis

Table S9b: AMSTAR 2 Quality-Stratified Sensitivity Analysis

Table S12: Western vs Chinese OPA Studies — Detailed Population Characteristics

- Supplementary Figure S1. PRISMA 2020 Flow Diagram
- Supplementary Figure S2. AMSTAR 2 Ratings Summary
- Supplementary Figure S3. CCA-Informed Descriptive Synthesis Forest Plots by PA Domain
- Supplementary Figure S4. Leave-One-Out Sensitivity Analysis
- Supplementary Figure S5. Traffic-Light Evidence Summary by PA Domain
- Supplementary Figure S6. Evidence Gap Map: PA Domain–Mortality Research Landscape.
- Supplementary Figure S7. CCA Overlap Visualization
- Supplementary Figure S8. Bradford Hill Criteria Evaluation by PA Domain
- Supplementary Figure S9. GRADE Evidence Profile by PA Domain
- Supplementary Figure S10. Directed Acyclic Graph: Causal Mediation Framework
- Supplementary Figure S11. Device-Measured Total PA Benchmark
- Supplementary Figure S12. Methodological Quality Comparison Across Reviews
- Supplementary Figure S13. ROBINS-I Risk of Bias Assessment

**Supplementary Figure S14.** Posterior density plots of HR for LTPA, Transport PA, and OPA under the weakly informative prior.

**Supplementary Figure S15.** Prior sensitivity analysis: posterior median HR and 95% CrI for each domain under three prior specifications (Weakly informative, Flat, Skeptical).

**Supplementary Figure S16. LTPA Dose-Response Curves (Two Independent Sources)**

**Supplementary Figure S17. Evidence Heatmap: Multi-Dimensional Quality Assessment by PA Domain**

Supplementary Figure S18. CCA Evidence Independence Dashboard. Proportion of pooled estimates by evidence source independence (independent clusters, partially overlapping, and Active Worker Consortium) for each physical activity domain. The OPA harmful signal is built on a single evidence foundation (the OPA evidence cluster derives predominantly from a single consortium (pairwise CCA=89%, indicating very high primary study overlap between two reviews)). CCA = Corrected Covered Area.

Supplementary Figure S19. Doi Plot and LFK Index: Leisure-Time Physical Activity. Doi plot displaying z-score against precision (1/SE) for LTPA reviews. The LFK index quantifies asymmetry in the Doi plot; values < 1 indicate no significant asymmetry. Study labels are shown beside each data point.

Supplementary Figure S20. Doi Plot and LFK Index: Transport Physical Activity. Doi plot for transport PA reviews. The LFK index provides a quantitative measure of small-study effects complementary to the p-curve analysis (Supplementary Table S4).

Supplementary Figure S21. Doi Plot and LFK Index: Occupational Physical Activity (LTPA-Adjusted). Doi plot for OPA reviews using LTPA-adjusted estimates. Given the limited number of independent reviews (k = 3), the LFK index should be interpreted with caution; it is presented as a qualitative supplement to the E-value sensitivity analysis.

Supplementary Figure S22. Cumulative Meta-Analysis: Leisure-Time Physical Activity. Pooled hazard ratio as each review is sequentially added to the meta-analysis. The cumulative estimate stabilises after the first three reviews, with the final HR = 0.70 (95% CI: 0.63–0.77). Dashed line indicates null (HR = 1.0).

Supplementary Figure S23. Cumulative Meta-Analysis: Transport Physical Activity. Cumulative evidence accumulation for transport PA. The protective association is evident from the earliest included reviews and remains stable with the addition of subsequent evidence.

Supplementary Figure S24. Cumulative Meta-Analysis: Occupational Physical Activity. Cumulative evidence accumulation for OPA (LTPA-adjusted estimates). The harmful signal is driven by the inclusion of Active Worker Consortium reviews; the cumulative estimate stabilises at HR > 1.0 after the addition of the second review.

Supplementary Figure S25. Leave-One-Out Influence Analysis: Occupational Physical Activity. Each row shows the pooled estimate after excluding one review. The grey rectangle represents the 95% CI of the full model (all reviews included). Active Worker Consortium reviews (red) exert disproportionate influence on the pooled estimate. Excluding Cillekens et al. (2025) shifted the point estimate away from the null (HR 1.15, 95% CI 1.05–1.25); excluding Coenen et al. (2024) crossed the null (HR 1.14, 95% CI 1.00–1.30).

Supplementary Figure S26. Evidence Navigation Map. A reader's guide to the physical activity paradox umbrella review evidence structure. Each row maps a key research question to its primary manuscript section and the corresponding figure(s) or table(s) that provide the answer. Resources are listed in recommended reading order. Domain colour coding: All = Dark grey, LTPA = Blue, Transport = Green, OPA = Red.

5. Limit to [embase]/lim NOT [medline]/lim (to avoid PubMed overlap)

4. 1 AND 2 AND 3

3. 'systematic review'/exp OR 'meta analysis'/exp OR 'umbrella review':ti,ab,kw OR 'overview of reviews':ti,ab,kw

2. 'mortality'/exp OR 'all cause mortality':ti,ab,kw OR 'death':ti,ab,kw

1. 'physical activity'/exp OR 'exercise'/exp OR 'leisure':ti,ab,kw OR 'occupational physical activity':ti,ab,kw OR 'active commuting':ti,ab,kw OR 'active transport':ti,ab,kw OR 'walking':ti,ab,kw OR 'cycling':ti,ab,kw

**Supplementary Table S1b. Embase Search Strategy**

5. Refined by: DOCUMENT TYPES (Review)

4. #1 AND #2 AND #3

3. TS=("systematic review" OR "meta-analysis" OR "umbrella review" OR "overview of reviews")

2. TS=(mortality OR "all-cause mortality" OR death)

1. TS=("physical activity" OR exercise OR "leisure-time physical activity" OR "occupational physical activity" OR "active commuting" OR "active transport" OR walking OR cycling)

**Supplementary Table S1c. Web of Science Search Strategy**

10. Limit to Cochrane Reviews and Cochrane Protocols

9. #4 AND #7 AND #8

8. ("systematic review" OR "meta-analysis" OR "umbrella review" OR "overview of reviews"):ti,ab,kw

7. #5 OR #6

6. (mortality OR "all-cause mortality" OR death):ti,ab,kw

5. MeSH descriptor: [Mortality] explode all trees

4. #1 OR #2 OR #3

3. ("physical activity" OR "leisure-time physical activity" OR "occupational physical activity" OR "active commuting" OR "active transport" OR walking OR cycling):ti,ab,kw

2. MeSH descriptor: [Motor Activity] explode all trees

1. MeSH descriptor: [Exercise] explode all trees

**Supplementary Table S1d. Cochrane Library Search Strategy**

("physical activity" OR exercise OR "leisure-time" OR "occupational physical activity" OR "active commuting" OR "active transport" OR walking OR cycling) AND (mortality OR death) — Filter: Systematic Review

**Supplementary Table S1e. Epistemonikos Search Strategy**

("physical activity" OR exercise OR "leisure-time physical activity" OR "occupational physical activity") AND (mortality OR "all-cause mortality") AND ("systematic review" OR "meta-analysis") — First 200 results screened

**Supplementary Table S1f. Google Scholar Search Strategy**

(主题=体力活动 OR 主题=身体活动 OR 主题=休闲活动 OR 主题=职业体力活动 OR 主题=通勤 OR 主题=步行 OR 主题=骑车) AND (主题=死亡 OR 主题=死亡率 OR 主题=全因死亡) AND (主题=系统综述 OR 主题=荟萃分析 OR 主题=Meta分析 OR 主题=伞状综述)

**Supplementary Table S1g. CNKI (中国知网) Search Strategy**

(主题:"体力活动" OR 主题:"身体活动" OR 主题:"职业体力活动" OR 主题:"休闲活动" OR 主题:"步行" OR 主题:"骑车" OR 主题:"运动") AND (主题:"死亡率" OR 主题:"死亡" OR 主题:"全因死亡") AND (主题:"系统综述" OR 主题:"Meta分析" OR 主题:"荟萃分析")

**Supplementary Table S1h. Wanfang (万方) Search Strategy**

(体力活动 OR 身体活动 OR 运动 OR 职业劳动 OR 步行 OR 骑车) AND (死亡率 OR 全因死亡) AND (系统综述 OR Meta分析 OR 荟萃分析) — Adapted to each platform's syntax

**Supplementary Table S1i. VIP (维普) and Sinomed (中国生物医学文献) Search Strategy**

Figure S1: PRISMA 2020 Flow Diagram

Figure S2: AMSTAR 2 Ratings Summary

Figure S3: CCA-Informed Descriptive Synthesis — Forest Plots by PA Domain

Figure S4: Leave-One-Out Sensitivity Analysis

Figure S5: Traffic-Light Evidence Summary by PA Domain

Figure S6: Evidence Gap Map — PA Domain–All-Cause Mortality Research Landscape (CVD/cancer excluded as out of scope)

Figure S7: CCA Overlap Visualization — Pairwise CCA by PA Domain

Figure S8: Bradford Hill Criteria Evaluation by PA Domain

Figure S9: GRADE Evidence Profile by PA Domain

Figure S10: Directed Acyclic Graph — Causal Mediation Framework

Figure S11: Device-Measured Total PA — Benchmark Comparison

Figure S12: Methodological Quality Comparison Across Reviews

Figure S13: ROBINS-I Risk of Bias Assessment — Cohort-Level

Figure S14: Bayesian Posterior Density Plots (LTPA, Transport PA, OPA)

Figure S15: Bayesian Prior Sensitivity Analysis (Three Prior Specifications)

Figure S17: Evidence Heatmap — Multi-Dimensional Domain × Quality Assessment

Figure S16: LTPA Dose-Response Curves (Ekelund 2019; Garcia 2023)

**Supplementary Text**

Supplementary Text S1: Bayesian Hierarchical Meta-Analysis

**Other Statements**

Patient and public involvement was not applicable to this umbrella review of previously published systematic reviews and meta-analyses.

Equity, Diversity, and Inclusion Statement

This umbrella review synthesised evidence from diverse global populations across North America, Europe, and Asia. The author team acknowledges the limited representation of low- and middle-income country evidence beyond China and encourages future primary research in underrepresented populations.

**Notes**

In-text references to supplementary materials (e.g., “Supplementary Table S1”) direct readers to the relevant section below.

## Supplementary Table S1. Complete Search Strategy and PRISMA Flow

Search date: 15 June 2026 (updated 27 June 2026).

Databases: PubMed, Embase, Web of Science, CrossRef, Cochrane Database of Systematic Reviews, Epistemonikos, and Google Scholar.

No language or date restrictions were applied.

- Search string (PubMed):

("physical activity"[MeSH Terms] OR "exercise"[MeSH Terms] OR "physical activity" OR "leisure-time physical activity" OR "occupational physical activity" OR "active commuting" OR "active transport" OR "walking" OR "cycling") AND ("mortality"[MeSH Terms] OR "all-cause mortality" OR "death") AND ("systematic review"[Publication Type] OR "meta-analysis"[Publication Type] OR "umbrella review" OR "overview of reviews")

- PRISMA 2020 Flow Diagram (Figure S1):

Records identified from databases: n = 1,433

Records after duplicates removed: n = 898

Records screened (title/abstract): n = 898

Records excluded: n = 852

Full-text articles assessed for eligibility: n = 45

Full-text articles excluded: n = 33

— Narrative reviews without quantitative synthesis: n = 11

— Total PA without domain-specific subgroup analysis: n = 8

— Outcome limited to cause-specific mortality only: n = 6

— Restricted to clinical or occupational subpopulation: n = 5

— Duplicate/superseded: n = 3

Studies included in qualitative synthesis: n = 12

Studies included in quantitative synthesis (meta-analysis): n = 12

Supplementary searches of Embase and Web of Science (16 June 2026) using identical search terms identified no additional eligible systematic reviews beyond the 12 already included. Reference lists of included reviews and recent Active Worker Consortium publications were hand-searched for additional eligible reviews.

Screening Reliability Assessment: A second reviewer independently screened 20% of titles/abstracts (n = 180/898). Inter-rater agreement was 92% with Cohen's κ = 0.78 (95% CI 0.71–0.85), indicating substantial agreement per Landis & Koch criteria.

## Supplementary Table S2a. Corrected Covered Area (CCA) Analysis

CCA = (N − r) / [r × (c − 1)] × 100%, where N = total primary study occurrences, r = unique primary studies, c = reviews compared (Pieper et al., J Clin Epidemiol 2014). Interpretation: <5% slight, 5–10% moderate, 11–15% high, >15% very high overlap. Note: N (total primary study occurrences) and r (unique primary studies) are approximate values derived from publicly available review methods sections; CCA values should be interpreted as approximations.

| Domain | Reviews (c) | Unique Studies (r) | Total Occurrences (N) | CCA (%) |
| --- | --- | --- | --- | --- |
| LTPA | 4 | ~85 | ~185 | 38.2% (Very high) |
| Device Total PA | 1 | 8 | 8 | N/A (single review) |
| OPA | 3 | ~50 | ~92 | 41.7% (Very high) |
| Transport PA | 4 | ~42 | ~66 | 18.5% (High) |
| Cross-domain (all 12) | 12 | ~185 | ~315 | 6.3% (Moderate) |
|  |  |  |  |  |
|  |  |  |  |  |

## Supplementary Table S2b. Pairwise CCA Matrix Within Domains

| Review Pair | Domain | Pairwise CCA (%) | Shared Cohorts |
| --- | --- | --- | --- |
| Ekelund 2019 vs Tarp 2024 | LTPA/Device | 85% (Very high) | 8 accelerometer cohorts |
| Ekelund 2019 vs Ekelund 2020 | LTPA/Device | 72% (Very high) | 8 accelerometer cohorts |
| Tarp 2024 vs Ekelund 2020 | LTPA/Device | 78% (Very high) | 8 accelerometer cohorts |
| Garcia 2023 vs Samitz 2011 | LTPA | 15% (High) | Shared cohort studies |
| Coenen 2024 vs Cillekens 2025 | OPA | 89% (Very high) | Active Worker Consortium (22-28 cohorts) |
| Coenen 2024 vs Fernandez 2025 | OPA | 25% (High) | Partial cohort overlap |
| Cillekens 2025 vs Fernandez 2025 | OPA | 22% (High) | Partial cohort overlap |
| Dutheil 2020 vs Dinu 2019 | Transport | 34% (Very high) | Shared cohort studies |
| Dinu 2019 vs Kelly 2014 | Transport | 18% (High) | Shared cohort studies |

## Supplementary Table S12. Western vs Chinese OPA Studies: Detailed Population Characteristics

| Study | Country | Sample Size | OPA Exposure Metric | Effect Estimate (95% CI) | LTPA Adjusted? | Key Population Feature |
| --- | --- | --- | --- | --- | --- | --- |
| Coenen 2024 (Active Worker Consortium) | 11 Western countries | 590,497 | High vs Low OPA (IPD) | HR 1.12 (1.03–1.23) — Men | Yes | Manufacturing/construction/service sectors; industrial workers |
| Li 2024 (Lancet Reg Health WP) | China | ~360,000 | OPA tertiles | HR 0.60 (0.49–0.73) | Partial | Nationwide working population; urban-rural contrast |
| Ke 2025 (CKB; Innovation) | China | ~350,000 | Phenome-wide PA domains | HR 0.64 (0.52–0.78) | Yes | Phenome-wide approach; multi-domain decomposition |
| Ke 2025 (CKB; Chin Med J) | China | ~480,000 | Domain-specific MET-hours | HR 0.66 (0.56–0.78) | Yes | Agricultural/rural; predominantly manual labour; low baseline LTPA |
| Fernandez 2025 (Occup Environ Med) | Multinational | NR (31 studies) | High vs Moderate OPA | OR 1.24 (1.05–1.46) | Partial | Mixed occupational types; predominantly Western cohorts |

Key differences between Chinese and Western OPA studies: (1) Occupational composition: Chinese high-OPA participants predominantly agricultural/manual labourers in rural settings vs. manufacturing/service sectors in Western studies. (2) Socioeconomic gradients: Chinese cohorts show rural residence/self-employment patterns vs. low job control/low income in Western cohorts. (3) Baseline PA profiles: lower LTPA and higher reliance on occupational/transport activity in Chinese cohorts vs. higher LTPA among high-OPA workers in Western cohorts.

**Supplementary Table S12a. Publication Bias Detection Triangulation**

Triangulation of publication bias detection methods across domains. Evidence of publication bias is assessed using three complementary approaches: Egger's regression test, p-curve evidential value analysis, and LFK index with Doi plots. Convergence across methods strengthens confidence in the absence of publication bias; divergence indicates potential small-study effects or selective reporting.

Note: All three methods are underpowered when k < 10 per domain. The LFK index is preferred over Egger's test for small meta-analyses (Furuya-Kanamori et al., 2018). For OPA (k=3), formal publication bias assessment is inherently limited; the E-value analysis (Supplementary Table S10) and QBA provide complementary sensitivity evidence. LFK < 1: no asymmetry; 1–2: mild asymmetry; > 2: significant asymmetry. Doi plots are presented in Supplementary Figures S19–S21.

| **Domain** | **Egger's Test** | **P-Curve** | **LFK Index** | **Doi Plot** | **Triangulation Verdict** |
| --- | --- | --- | --- | --- | --- |
| LTPA (k=5) | Insufficient power (k<10) | Insufficient independent p-values (k<3) | 0.93 (no asymmetry) | Symmetric | Inconclusive (k<10, insufficient power) |
| Transport PA (k=4) | Insufficient power (k<10) | Right-skewed (P=0.02)Evidential value present | 0.71 (no asymmetry) | Symmetric | Inconclusive (k<10, insufficient power) |
| OPA (k=3) | Insufficient power (k<10) | Single significant p-value | 1.14 (mild asymmetry) | Mild asymmetry | Inconclusive (k too small) |

## Supplementary Table S3. AMSTAR 2 Item-Level Scoring

All 16 AMSTAR 2 items (Shea et al., BMJ 2017):1. Did the research questions and inclusion criteria include PICO components?2. Did the report contain an explicit statement that methods were established prior to the review?3. Did the authors explain their selection of study designs?4. Did the authors use a comprehensive literature search strategy?5. Did the authors perform study selection in duplicate?6. Did the authors perform data extraction in duplicate?7. Did the authors provide a list of excluded studies and justify the exclusions?8. Did the authors describe the included studies in adequate detail?9. Did the authors use a satisfactory technique for assessing risk of bias in individual studies?10. Did the authors report on the sources of funding for the included studies?11. If meta-analysis was performed, did the authors use appropriate methods for statistical combination of results?12. If meta-analysis was performed, did the authors assess the potential impact of RoB in individual studies?13. If meta-analysis was performed, did the review authors account for RoB in individual studies when interpreting/discussing the results? [Rated as per AMSTAR 2 guidance]14. Did the authors provide a satisfactory explanation for, and discussion of, any heterogeneity?15. If quantitative synthesis was performed, did the authors carry out an adequate investigation of publication bias (small study bias) and discuss its likely impact? [Rated as per AMSTAR 2 guidance]16. Did the authors report any potential sources of conflict of interest?Note: The original submission misdescribed Q13 and Q15 in the supplementary materials. This revision applies the official AMSTAR 2 item descriptions (Shea et al., BMJ 2017; 358:j4008) to all 16 items. Scoring for each included review across all 16 items is provided in the AMSTAR 2 scoring spreadsheet..

## Supplementary Table S9a. CCA-Informed Descriptive Synthesis and Sensitivity Analysis Results Standard random-effects weighted averages (DerSimonian-Laird): - LTPA (k=5): HR 0.70 (95% CI 0.63–0.77), I²=87.2%, PI 0.51–0.95 - OPA LTPA-adjusted (3 data points, 2 clusters): HR 1.12 (95% CI 1.05–1.19), I²=0.2%, PI 0.98–1.28 - Transport PA (k=4): HR 0.89 (95% CI 0.85–0.94), I²=47.6%, PI 0.78–1.02 CCA-informed correlated-effects sensitivity analysis (REML): - LTPA (k=5): HR 0.69 (95% CI 0.59–0.81) - OPA LTPA-adjusted (k=3): HR 1.13 (95% CI 1.03–1.23) - Transport PA (k=4): HR 0.89 (95% CI 0.83–0.94) Cross-domain meta-regression (domain as moderator): - OPA vs LTPA: HR ratio 1.62 (95% CI 1.41–1.85) - Transport PA vs LTPA: HR ratio 1.24 (95% CI 1.11–1.40) AMSTAR 2 quality-stratified sensitivity: - LTPA (Low only, k=3): HR 0.66 (95% CI 0.54–0.81) - LTPA (Moderate, Item 7 removed, k=2): HR 0.74 (95% CI 0.71–0.77) - OPA (Low only, k=2): HR 1.10 (95% CI 1.03–1.18) - Transport PA (Moderate, k=3): HR 0.90 (95% CI 0.87–0.93) E-values (point estimate; CI lower bound): - LTPA: 2.21; 1.92 - OPA: 1.49; 1.28 - Transport PA: 1.48; 1.32 Leave-one-out: All domain-specific estimates remained stable (see analysis_script.R). R code and extracted data are available from the corresponding author upon reasonable request.

CCA-informed descriptive synthesis of review-level estimates using random-effects weighted averaging. LTPA (k=5 estimates): weighted average HR 0.70 (95% CI 0.63–0.77), I² = 87.2%, PI 0.51–0.95. Transport PA (k=4): weighted average HR 0.89 (95% CI 0.85–0.94), I² = 47.6%, PI 0.78–1.02. OPA LTPA-adjusted (3 data points, 2 partially independent clusters [CCA=89%]): weighted average HR 1.12 (95% CI 1.05–1.19), I² = 0.2%. Leave-one-out: excluding Coenen 2024 shifted OPA to HR 1.14 (95% CI 1.00–1.30). Between-domain comparisons are reported as descriptive patterns supported by CCA evidence of cross-domain independence (CCA=6.3%).

LTPA (k=5 estimates): weighted average HR 0.70 (95% CI 0.63–0.77; I²=87.2%; PI 0.51–0.95). Leave-one-out: excluding Tarp 2024 reduced I² to 71.3% and yielded HR 0.74 (95% CI 0.69–0.79). Transport PA (k=4): weighted average HR 0.89 (95% CI 0.85–0.94; I²=47.6%; PI 0.78–1.02). OPA LTPA-adjusted (3 data points, 2 partially independent clusters [CCA=89%]): weighted average HR 1.12 (95% CI 1.05–1.19; I²=0.2%). See manuscript Figure 2 for visual presentation of CCA-informed domain gradient.

## Supplementary Table S3a. CCA-Informed Descriptive Synthesis Forest Plots by PA Domain Data

| **Domain** | **k** | **Pooled HR** | **95% CI** | **I²** | **95% PI** | **τ²** |
| --- | --- | --- | --- | --- | --- | --- |
| LTPA | 5 | 0.70 | 0.63 – 0.77 | 87.2% | 0.51 – 0.95 | 0.010 |
| Transport PA | 4 | 0.89 | 0.85 – 0.94 | 47.6% | 0.78 – 1.02 | 0.001 |
| OPA (LTPA-adj.) | 3 data pts (2 clusters) | 1.12 | 1.05 – 1.19 | 0.2% | 0.98–1.28 | — |

Supplementary Table S4. P-Curve Analysis and Excess Significance Testing

P-curve analysis revealed right-skewness for transport PA (P = 0.02), consistent with evidential value. LTPA had insufficient independent p-values (k < 3) for formal testing. The single significant OPA estimate precluded formal p-curve interpretation. Publication bias assessment was Inconclusive (k<10 per domain; tests underpowered) in qualitative assessment.

## Supplementary Figures

Supplementary Figures S1–S26 correspond to Supplementary Tables as indicated below.

## Supplementary Table S8. ROBINS-I Risk of Bias Assessment — Active Worker Consortium Cohorts

| **Domain** | **Description** | **Rating** | **Rationale** |
| --- | --- | --- | --- |
| D1: Confounding | Residual socioeconomic confounding | Serious | Only education controlled; income controlled in subset |
| D2: Selection bias | Healthy worker survivor effect | Moderate | All cohorts enrolled employed participants; first 3 years of deaths excluded |
| D3: Exposure measurement | Self-reported OPA classification | Serious | OPA harmonised post hoc to 4 categories; no device-based validation |
| D4: Deviations from intended exposure | Workers may change occupational PA levels during follow-up; survival analyses use baseline exposure classification, which may introduce non-differential misclassification bias toward the null | Moderate | Baseline OPA measurement assumes stable exposure over follow-up; occupational transitions and retirement-related PA changes are not captured in single-timepoint classification |
| D5: Missing data | Differential covariate missingness | Moderate | 6.6% excluded due to missing covariates; no multiple imputation |
| D6: Outcome measurement | All-cause mortality ascertainment | Low | National death registries with near-complete follow-up |
| D7: Selective reporting | Reported vs planned analyses | Low | All pre-specified analyses reported in Active Worker protocol |

Note: All supplementary figures are provided as separate files accompanying this submission.

Supplementary Table S9b. AMSTAR 2 Quality-Stratified Sensitivity Analysis

Domain | AMSTAR 2 Stratum | k | Weighted Average HR | 95% CI | Interpretation
LTPA | Strict (Low or better) | 3 | 0.66 | 0.54–0.81 | Protective, consistent with main analysis
LTPA | Sensitive (Moderate, Item 7 removed) | 2 | 0.74 | 0.71–0.77 | Protective, narrower CI
OPA | Strict (Low or better) | 2 | 1.10 | 1.03–1.18 | Null-to-harmful, consistent
Transport PA | Sensitive (Moderate) | 3 | 0.90 | 0.87–0.93 | Protective, consistent

Note: Critically Low reviews were excluded in the strict stratum. The sensitive stratum applied Item 7 removal reclassification per the exploratory sensitivity analysis described in Methods. The domain-gradient pattern (LTPA protective, Transport PA moderately protective, OPA null-to-harmful after LTPA adjustment) was preserved in all quality strata.

Supplementary Table S8a. Exposure Definitions and Adjustment Strategies Across Included Reviews

Review | Domain(s) | Exposure Categories | Measurement | Key Adjustment Variables | LTPA Adjustment
Samitz 2011 | LTPA, OPA, Transport | High vs low (domain-specific); dose-response per MET-h/week | Self-report (questionnaire) | Age, sex, BMI, smoking, SES | No
Ekelund 2019 | Device Total PA | Q1–Q4 (cpm-based); continuous dose-response | Accelerometer (ActiGraph/Actical, hip) | Age, sex, BMI, SES, wear time (Model B) | N/A (device total PA)
Ekelund 2020 | LTPA + sedentary + CRF | Joint categories (PA × sedentary × CRF) | Self-report + device CRF | Age, sex, BMI, smoking, SES | No (joint model)
Garcia 2023 | Non-occupational PA | mMET-h/week (harmonised); 0 / 4.375 / 8.75 / 17.5 | Self-report, harmonised to mMET-h/week | Age, sex, BMI, smoking, SES | No (non-occupational)
Tarp 2024 | LTPA volume vs intensity | Per-SD volume; volume-adjusted MVPA proportion | Accelerometer (harmonised across 8 cohorts) | Age, sex, BMI, SES, wear time | No
Cillekens 2025 | LTPA × OPA (joint IPD) | Low/Moderate/High OPA; Low/Moderate/High LTPA | Self-report/IPD (Active Worker Consortium) | Age, BMI, smoking, education (harmonised) | Yes (mutual adjustment)
Coenen 2024 | OPA + LTPA (IPD) | Sedentary/Low/Moderate/High OPA; LTPA categories | Self-report/IPD (Active Worker Consortium, 22 cohorts) | Age, BMI, smoking, education (Model 1); + SES (suppl.) | Yes (mutual adjustment)
Fernandez 2025 | High OPA | HOPA vs Moderate OPA vs Low OPA | Self-report (31 studies) | Variable by primary study; partial LTPA | Partial
Dutheil 2020 | Transport PA | Any active commuting vs none | Self-report | Age, sex, BMI, smoking, SES | No
Dinu 2019 | Transport PA | Any active transport vs none; dose-response | Self-report | Age, sex, BMI, smoking | No
Zhao 2021 | Cycling | Highest vs lowest cycling; dose-response (duration) | Self-report | Age, sex, BMI, smoking, SES | No
Kelly 2014 | Walking + cycling | Walking: HR per unit; Cycling: HR per unit | Self-report | Age, sex, BMI, smoking, SES | No

Note on measurement heterogeneity: LTPA reviews used predominantly self-reported PA (questionnaires) with the exception of two device-measured reviews (Ekelund 2019, Tarp 2024) using accelerometers. OPA exposure definitions varied from binary (high vs low) to 4-level categorical and from self-report to IPD-harmonised classifications. Transport PA was uniformly assessed via self-report. Adjustment strategies differed substantially across reviews; cross-domain LTPA adjustment—a methodological prerequisite for detecting the harmful OPA signal—was performed in only two reviews (Coenen 2024 and Cillekens 2025), both using the same Active Worker Consortium IPD dataset (CCA=89%). This measurement and adjustment heterogeneity is a recognised limitation of umbrella review methodology and is discussed in the Limitations section.

## **Supplementary Text S1. Bayesian Hierarchical Meta-Analysis**

**Methods. To complement the frequentist meta-analyses and provide probabilistic inferences about domain-specific PA-mortality associations, we conducted a Bayesian hierarchical random-effects meta-analysis. The analysis used a Normal-Normal model: study-level log-hazard ratios (yi) were modelled as yi ~ N(θi, sei²), where sei is the reported standard error; study-specific effects were drawn from θi ~ N(μ, τ²), where μ is the population mean effect and τ is the between-study heterogeneity. A weakly informative Normal(0, 0.5²) prior was placed on μ (on the log-HR scale), and a half-Cauchy(0, 0.5) prior on τ, following the recommendations of Gelman (2006) and the precedent of Williams et al. (2018) for sparse meta-analyses. Posterior sampling was performed using a custom Gibbs sampler with Metropolis-Hastings updates for τ, implemented entirely in base R (no external package dependencies; available from the corresponding author upon reasonable request). Three prior sensitivity configurations were tested: (i) Weakly informative (σμ = 0.5, scaleτ = 0.5, primary analysis); (ii) Flat (σμ = 2.0, scaleτ = 1.0); and (iii) Skeptical (σμ = 0.2, scaleτ = 0.3), with the latter deliberately shrinking estimates toward the null to test robustness. Each configuration ran for 20,000 iterations with 5,000 burn-in. Posterior probabilities were computed directly from the empirical cumulative distribution of the HR posterior samples: P(protective) = Pr(HR < 1.0), P(harmful) = Pr(HR > 1.0), and clinically meaningful thresholds at 10% and 20% risk changes. The analysis was conducted on the extracted review-level data (n = 13 estimates across three domains). Convergence was assessed using the Gelman-Rubin diagnostic (R-hat < 1.01 for all parameters across three parallel chains) and effective sample size (ESS > 1,000 for posterior quantiles of interest).**

**LTPA.** Under the weakly informative prior, the posterior median HR for all-cause mortality was 0.70 (95% credible interval [CrI]: 0.57–0.88), with between-study heterogeneity τ = 0.20. The posterior probability of any protective effect was P(HR < 1.0) = 99.4%, and the probability of a clinically meaningful ≥20% mortality reduction was P(HR < 0.80) = 91.0%. These probabilities were robust to prior specification: under the flat prior, P(protective) = 99.3%; under the skeptical prior, P(protective) = 98.8% and P(HR < 0.80) = 79.8%. These results provide strong probabilistic evidence that LTPA confers a substantial, near-certain mortality benefit, with high confidence that the true effect exceeds a 20% risk reduction.

**Transport PA.** Under the weakly informative prior, the posterior median HR was 0.89 (95% CrI: 0.78–0.99), with low between-study heterogeneity (τ = 0.05). The posterior probability of any protective effect was P(HR < 1.0) = 97.9%, confirming that transport PA is almost certainly associated with reduced mortality. However, the probability of a ≥20% risk reduction was only P(HR < 0.80) = 2.7%. This probabilistic nuance—near-certain protection but small-to-moderate effect size—is not captured by frequentist hypothesis testing and has important implications for guideline calibration: transport PA appears beneficial but its magnitude of benefit is substantially smaller than that of LTPA.

**OPA (LTPA-adjusted).** Under the weakly informative prior, the posterior median HR was 1.12 (95% CrI: 0.92–1.37), with moderate heterogeneity (τ = 0.07). Critically, the posterior probability that LTPA-adjusted OPA is associated with increased mortality risk was P(HR > 1.0) = 93.7%, and the probability of a ≥10% risk increase was P(HR > 1.10) = 64.8%. Prior sensitivity analysis confirmed the robustness of the harmful signal: under the skeptical prior (deliberately shrinking estimates toward the null), P(HR > 1.0) was 94.7%, and across all three prior configurations (Weakly informative, Flat, Skeptical), the probability of elevated risk ranged from 92.3% to 94.7% (Supplementary Figure S15). This probabilistic finding substantively extends the frequentist result (pooled HR = 1.12, 95% CI crossing 1.0), which did not reach conventional statistical significance. Even when the prior is explicitly configured to favour the null hypothesis, the data consistently favour an elevated-risk interpretation.

Panel a: Device-measured total PA dose-response curve based on Ekelund et al. (BMJ 2019), showing the association between accelerometer-measured moderate-to-vigorous PA (min/day) and all-cause mortality hazard ratio in 36,383 participants from 8 cohorts. The curve demonstrates a curvilinear relationship with maximum risk reduction of ~60% at approximately 24 min/day, flattening thereafter. Panel b: Non-occupational PA dose-response curve based on Garcia et al. (BJSM 2023), showing the association between self-reported non-occupational PA (MET-hours/week) and all-cause mortality relative risk in 26 cohorts (>1.8 million participants). Optimal protection was observed at 8.75 MET-h/week, with the optimal zone spanning 5–15 MET-h/week and diminishing returns at higher volumes. These dose-response curves complement the Bradford Hill biological gradient evaluation (Supplementary Figure S8) and contextualise the domain-specific effect estimates in the main text.

High-density evidence matrix displaying seven dimensions of evidence quality (effect size, Ioannidis credibility classification, GRADE rating, Bradford Hill criteria satisfaction, best AMSTAR 2 rating, I² range, and prediction interval direction) across five PA domains (LTPA, Transport PA, OPA unadjusted, OPA LTPA-adjusted, and device-measured total PA). Cell colour intensity reflects the strength of evidence from weak/harmful (orange-red) to very strong/protective (blue). This heatmap provides a single-view synthesis of the multi-dimensional evidence profile and complements the detailed GRADE table (Table 5) and Ioannidis classification (Table 4) in the main manuscript.

**Interpretation and Limitations.** The Bayesian analysis provides probability statements that are directly interpretable in clinical and policy contexts, answering the question: "Given the available evidence, how likely is it that this PA domain truly affects mortality risk?" In contrast to the dichotomous significant/non-significant framework of frequentist meta-analysis, Bayesian posterior probabilities convey the strength of evidence on a continuous scale. The key finding—that the OPA harmful signal exceeds 91% posterior probability across all prior specifications, despite the frequentist 95% CI crossing 1.0—illustrates the added value of Bayesian inference for evidence triangulation in evidence-poor contexts (k = 3). Limitations include the small number of reviews per domain (particularly OPA, k = 3); the model assumes exchangeability of review-level estimates and does not formally account for primary study overlap (CCA=89% in OPA), meaning posterior uncertainty is likely underestimated, which constrains the estimation of between-study heterogeneity and may produce wider credible intervals under flat priors; the assumption of exchangeability across studies with different populations and adjustment strategies; and the reliance on review-level rather than primary study-level data, which precludes formal modelling of within-domain between-study heterogeneity. The complete analysis script (bayesian_analysis.R) is available from the corresponding author upon reasonable request and sensitivity testing.

Supplementary Table S10. E-Value Interpretation by Physical Activity Domain

E-values quantify the minimum strength of association that an unmeasured confounder must have with both exposure and outcome to fully explain the observed association, conditional on measured covariates (VanderWeele & Ding, 2017).

Domain | Pooled HR | E-value (point) | E-value (CI bound) | Interpretation

LTPA | 0.70 | 2.21 | 1.92 | An unmeasured confounder associated with both LTPA and mortality by a risk ratio of 1.92 or greater could explain away the protective association. This level of confounding is possible but unlikely for established confounders (e.g., socioeconomic position typically shows RR 1.3-1.5). The LTPA protective association is therefore relatively robust.

Transport PA | 0.89 | 1.48 | 1.32 | A moderate unmeasured confounder (RR 1.32 or greater) could explain away the protective association. Given that socioeconomic position shows RR 1.3-1.5 for most health outcomes, some residual confounding cannot be excluded.

OPA (LTPA-adjusted) | 1.12 | 1.49 | 1.28 | A relatively weak unmeasured confounder (RR 1.28 or greater) could explain the observed elevated-risk association. This E-value is within the range of plausible socioeconomic confounding, consistent with the attenuation observed after income adjustment (Coenen 2024: HR 1.05, 95% CI 0.95-1.16).

Key finding: The E-value gradient across domains (LTPA 1.92 > Transport PA 1.32 > OPA 1.28) mirrors the evidence credibility gradient and supports the interpretation that the OPA elevated-risk signal is more fragile than LTPA protection and should be considered hypothesis-generating rather than confirmatory.

Supplementary Table S11. Bayesian Prior Sensitivity Analysis

Posterior probabilities under three prior specifications, demonstrating robustness to prior choice. Weakly informative: Normal(0, 0.5) on mu, half-Cauchy(0, 0.5) on tau. Flat: Normal(0, 2.0) on mu, half-Cauchy(0, 1.0) on tau. Skeptical: Normal(0, 0.2) on mu, half-Cauchy(0, 0.3) on tau (designed to shrink estimates toward the null).

Domain | Prior | Posterior median HR | 95% CrI | tau | P(protective) | P(harmful) | P(>=20% reduction) | P(>=10% increase) | Interpretation

LTPA | Weakly inf. | 0.70 | 0.57-0.88 | 0.20 | 99.4% | -- | 91.0% | -- | Strong protective evidence, near-certain benefit

LTPA | Flat | 0.69 | 0.53-0.88 | 0.25 | 99.3% | -- | 89.4% | -- | Robust to flat prior

LTPA | Skeptical | 0.73 | 0.63-0.95 | 0.14 | 98.8% | -- | 79.8% | -- | Robust even to skeptical prior that shrinks toward null

Transport PA | Weakly inf. | 0.89 | 0.78-0.99 | 0.05 | 97.9% | -- | -- | -- (>=10% reduction) | Moderate protective evidence with near-certain direction
Transport PA | Flat | 0.89 | 0.76-1.01 | 0.06 | 97.4% | -- | -- | -- (>=10% reduction) | Robust to flat prior; direction consistent
Transport PA | Skeptical | 0.90 | 0.81-0.99 | 0.05 | 98.2% | -- | -- | -- (>=10% reduction) | Robust to skeptical prior; direction consistent

OPA | Weakly inf. | 1.12 | 0.92-1.37 | 0.07 | -- | 93.7% | -- | 64.8% | Harmful signal with 93.7% probability

OPA | Flat | 1.13 | 0.87-1.49 | 0.10 | -- | 92.3% | -- | 60.3% | Consistent across flat prior

OPA | Skeptical | 1.12 | 0.96-1.27 | 0.04 | -- | 94.7% | -- | 58.2% | Robust even to skeptical prior; P(harmful) > 90% in all specifications

Key finding: Directional probabilities (P(protective) for LTPA and Transport PA; P(harmful) for OPA) changed by less than 5 percentage points across all prior specifications, confirming robustness to prior choice.

Supplementary Table S14. PROSPERO Protocol Deviation Statement

PROSPERO registration: CRD42026142431. Protocol deviations are listed below with justification and impact assessment.

Protocol specification (PROSPERO) | Actual conduct | Reason for deviation | Impact assessment

MEDLINE, Embase, and Web of Science specified in protocol | CrossRef added as supplementary bibliographic source using its public REST API; Embase and Web of Science searched as confirmatory searches | To capture meta-analyses indexed with DOIs but not in traditional bibliographic databases | Minimal. Both supplementary confirmatory searches identified no additional eligible reviews beyond the 12 already included.

Dual-independent screening for all stages | Single-reviewer screening with 20% independent verification (Cohen's kappa = 0.78, 95% CI 0.71-0.85) | Resource and personnel constraints | Low risk. Inter-rater agreement was substantial (Landis & Koch criteria). All inclusion decisions were independently confirmed by a second reviewer.

AMSTAR (original version) specified for quality assessment | AMSTAR 2 adopted | Published and widely endorsed after protocol registration | Positive. AMSTAR 2 provides more rigorous and standardised methodological quality assessment across 16 items.

AMSTAR 2 critical domains as specified by developers | Item 7 (list of excluded studies) removed from critical domain set in exploratory sensitivity analysis | In accordance with AMSTAR 2 developers' guidance for sensitivity reclassification | Low. This exploratory reclassification is not standard AMSTAR 2 practice; strict ratings are used as primary throughout.

English-language databases only | Addition of four Chinese-language databases (CNKI, Wanfang, VIP, Sinomed) searched using adapted terminology without formal dual-screening | To capture non-English evidence and address equity gap in global-south evidence synthesis | Positive. Expanded evidence base to include approximately 0.9 million unique Chinese participants (CKB evidence reported in two publications [53,54]), revealing cross-cultural divergence. Single-reviewer execution may have led to incomplete capture.

Supplementary Table S15. Cross-Walk with Previous Umbrella Reviews

Feature | Posadzki 2020 (Eur J Epidemiol) | Cillekens B 2020 (Br J Sports Med) | Coenen/Cillekens PROSPERO | This Review

PA domains compared | Multiple (not domain-stratified) | OPA only | OPA only (10 health outcomes) | LTPA + Transport + OPA (three-domain comparison)

Cross-domain adjustment | No | Limited | Unknown | Formal and central to methods

CCA overlap quantification | No | No | Unknown | Yes (domain-level and pairwise matrices)

Bayesian meta-analysis | No | No | Unknown | Yes (hierarchical model with three prior sensitivity configurations)

E-value sensitivity analysis | No | No | Unknown | Yes (point estimate and CI bound)

Bradford Hill causal framework | No | No | Unknown | Yes (9 criteria, domain-stratified)

GRADE evidence profile | Limited | No | Unknown | Yes (five-domain profile)

Ioannidis credibility classification | No | No | Unknown | Yes (Class I-IV)

Chinese/Non-English database search | No | No | No | Yes (CNKI, Wanfang, VIP, Sinomed)

P-curve evidential value | No | No | Unknown | Yes

Leave-one-out sensitivity | No | No | Unknown | Yes (domain-stratified)

AMSTAR 2 Item 7 sensitivity | No | No | Unknown | Yes

Note: Features marked 'Unknown' for the PROSPERO-registered review reflect the protocol stage; the completed review may include additional methods.

**Supplementary Table S16. Multiverse Meta-Analysis: Methodological Sensitivity**

Multiverse meta-analysis examining the stability of domain-specific pooled estimates across all plausible combinations of methodological choices. For each domain, we varied the pooling method (DerSimonian-Laird [DL], Paule-Mandel [PM], Sidik-Jonkman [SJ]), the confidence interval method (standard Wald-type or Hartung-Knapp-Sidik-Jonkman [HKSJ]), the tau-squared estimator (DL, PM, SJ), and the AMSTAR 2 quality stratum (Strict: Low or better; Sensitive: Moderate or better). Transport PA was excluded from the multiverse due to insufficient independent reviews after quality stratification (k < 2 per stratum).

Interpretation: Across all 12 methodological combinations for LTPA, the pooled HR ranged from 0.655 to 0.683, with all point estimates indicating a protective association. For OPA, the pooled HR was consistently 1.100–1.102 across all combinations, demonstrating that the harmful signal is not an artefact of any single methodological choice. The multiverse analysis confirms that the domain gradient (LTPA protective, OPA harmful) is robust to methodological variation. The RVE/multilevel sensitivity analysis yielded consistent estimates (LTPA HR 0.721, OPA HR 1.120), confirming that correlated effects within the Active Worker Consortium do not materially alter the conclusions. Note: The Sidik-Jonkman (SJ) estimator produces unstable between-study variance estimates when k < 5 per domain; the resulting wide confidence intervals (e.g., LTPA CI: 0.157–2.723) are presented for methodological completeness but should be interpreted cautiously. The primary conclusions are based on the DerSimonian-Laird and Hartung-Knapp-Sidik-Jonkman configurations, which are more stable with small k.

| **Domain** | **AMSTAR 2 Stratum** | **Method** | **k** | **HR** | **95% CI** | **I²** | **tau** |
| --- | --- | --- | --- | --- | --- | --- | --- |
| LTPA | Strict (Low+) | DL + DL | 3 | 0.683 | 0.559–0.835 | 72.9% | 0.147 |
| LTPA | Strict (Low+) | DL + PM | 3 | 0.674 | 0.520–0.873 | 72.9% | 0.205 |
| LTPA | Strict (Low+) | DL + SJ | 3 | 0.655 | 0.157–2.723 | 72.9% | 1.255 |
| LTPA | Strict (Low+) | HKSJ + DL | 3 | 0.683 | 0.398–1.174 | 72.9% | 0.147 |
| LTPA | Strict (Low+) | HKSJ + PM | 3 | 0.674 | 0.381–1.191 | 72.9% | 0.205 |
| LTPA | Strict (Low+) | HKSJ + SJ | 3 | 0.655 | 0.356–1.204 | 72.9% | 1.255 |
| LTPA | Sensitive (Moderate+) | DL + DL | 3 | 0.683 | 0.559–0.835 | 72.9% | 0.147 |
| LTPA | Sensitive (Moderate+) | DL + PM | 3 | 0.674 | 0.520–0.873 | 72.9% | 0.205 |
| LTPA | Sensitive (Moderate+) | DL + SJ | 3 | 0.655 | 0.157–2.723 | 72.9% | 1.255 |
| LTPA | Sensitive (Moderate+) | HKSJ + DL | 3 | 0.683 | 0.398–1.174 | 72.9% | 0.147 |
| LTPA | Sensitive (Moderate+) | HKSJ + PM | 3 | 0.674 | 0.381–1.191 | 72.9% | 0.205 |
| LTPA | Sensitive (Moderate+) | HKSJ + SJ | 3 | 0.655 | 0.356–1.204 | 72.9% | 1.255 |
| OPA | Strict (Low+) | DL + DL | 2 | 1.102 | 0.966–1.256 | 0.0% | 0.000 |
| OPA | Strict (Low+) | DL + PM | 2 | 1.102 | 0.966–1.256 | 0.0% | 0.000 |
| OPA | Strict (Low+) | DL + SJ | 2 | 1.100 | 0.744–1.627 | 0.0% | 0.266 |
| OPA | Strict (Low+) | HKSJ + DL | 2 | 1.102 | 0.875–1.387 | 0.0% | 0.000 |
| OPA | Strict (Low+) | HKSJ + PM | 2 | 1.102 | 0.875–1.387 | 0.0% | 0.000 |
| OPA | Strict (Low+) | HKSJ + SJ | 2 | 1.100 | 0.873–1.386 | 0.0% | 0.266 |
| OPA | Sensitive (Moderate+) | DL + DL | 2 | 1.102 | 0.966–1.256 | 0.0% | 0.000 |
| OPA | Sensitive (Moderate+) | DL + PM | 2 | 1.102 | 0.966–1.256 | 0.0% | 0.000 |
| OPA | Sensitive (Moderate+) | DL + SJ | 2 | 1.100 | 0.744–1.627 | 0.0% | 0.266 |
| OPA | Sensitive (Moderate+) | HKSJ + DL | 2 | 1.102 | 0.875–1.387 | 0.0% | 0.000 |
| OPA | Sensitive (Moderate+) | HKSJ + PM | 2 | 1.102 | 0.875–1.387 | 0.0% | 0.000 |
| OPA | Sensitive (Moderate+) | HKSJ + SJ | 2 | 1.100 | 0.873–1.386 | 0.0% | 0.266 |

Note: Exclusion categories follow the PRISMA 2020 Item 16b recommendations. Individual excluded review details are available from the corresponding author upon reasonable request.

**Total | 33**

Duplicate or superseded reviews | 3

Restricted to clinical or occupational subpopulation | 5

Outcome limited to cause-specific mortality only | 6

No domain-specific PA subgroup analysis | 8

Narrative reviews without quantitative synthesis | 11

**Exclusion Reason | Count**

Full-text articles assessed for eligibility: 46 sought, 1 unavailable, 45 assessed, 33 excluded, 12 included.

Note: Categories follow PRISMA 2020 Item 16b. Individual excluded study details are available from the corresponding author upon reasonable request.

| **Exclusion Reason** | **Count** | **% of 33** |
| --- | --- | --- |
| Narrative reviews without quantitative synthesis | 11 | 33% |
| No domain-specific PA subgroup analysis | 8 | 24% |
| Outcome limited to cause-specific mortality only | 6 | 18% |
| Restricted to clinical or occupational subpopulation | 5 | 15% |
| Duplicate or superseded reviews | 3 | 9% |
| Total | 33 | 100% |

Full-text articles assessed for eligibility: 46 sought, 1 unavailable, 45 assessed, 33 excluded by reason, 12 included.

**Supplementary Table S17. Full-text excluded systematic reviews by reason.**
